# Case Fatality Rate of Enteric Fever in Endemic Countries: A Systematic Review and Meta-analysis

**DOI:** 10.1093/cid/ciy190

**Published:** 2018-03-07

**Authors:** Zoë Pieters, Neil J Saad, Marina Antillón, Virginia E Pitzer, Joke Bilcke

**Affiliations:** 1 Vaccine & Infectious Disease Institute, University of Antwerp, Wilrijk; 2 Department of Mathematics and Statistics, Hasselt University, Diepenbeek, Belgium; 3 Department of Epidemiology of Microbial Diseases, Yale School of Public Health, New Haven, Connecticut

**Keywords:** typhoid fever, *Salmonella enterica* serovar Typhi, *Salmonella enterica* serovar Paratyphi, mortality, antimicrobial resistance

## Abstract

Enteric fever is a febrile illness, occurring mostly in Asia and Africa, which can present as a severe and possibly fatal disease. Currently, a case fatality rate (CFR) of 1% is assumed when evaluating the global burden of enteric fever. Until now, no meta-analysis has been conducted to summarize mortality from enteric fever. Therefore, we conducted a systematic review and meta-analysis to aggregate all available evidence. We estimated an overall CFR of 2.49% (95% confidence interval, 1.65%–3.75%; n = 44), and a CFR in hospitalized patients of 4.45% (2.85%–6.88%; n = 21 of 44). There was considerably heterogeneity in estimates of the CFR from individual studies. Neither age nor antimicrobial resistance were significant prognostic factors, but limited data were available for these analyses. The combined estimate of the CFR for enteric fever is higher than previously estimated, and the evaluation of prognostic factors, including antimicrobial resistance, urgently requires more data.

## Teaser

We conducted a systematic literature review and meta-analysis and estimated the case fatality rate of enteric fever to be 2.49% (95% confidence interval, 1.65%–3.75%; n = 44), 2.5 times higher than what was assumed up until now.

Enteric (typhoid and paratyphoid) fever is caused by the bacteria *Salmonella enterica* serovars Typhi (*S*. Typhi) and Paratyphi A, B, and C (*S*. Paratyphi), which exclusively infect humans and are transmitted through the ingestion of contaminated food or water [1, 2]. Illness lasts 3–21 days on average and can be severe and possibly fatal [3, 4]. Infected individuals often present with high temperature, as well as abdominal discomfort with possible vomiting, and headache, and complications include neurologic involvement, intestinal perforation, and death [1, 4]. The burden of enteric fever mostly occurs in Africa and Asia, and globally the disease is estimated to cause about 17.8 million cases (95% confidence interval [CI], 6.9–48.4) and 129000 deaths (95% CI: 75000–208000) annually [5–8].

Morbidity and mortality rates due to enteric fever may increase further in light of rising antimicrobial resistance (AMR). Antimicrobial therapy was first introduced in 1948, with chloramphenicol-resistant isolates emerging within 2 years after its introduction [9, 10]. In the 1980s, continued and inappropriate use of ampicillin, chloramphenicol, and cotrimoxazole resulted in the emergence of multidrug-resistant strains of *S*. Typhi, which exhibit simultaneous resistance to all 3 antibiotics [11]. Currently, fluoroquinolones are the preferred treatment option, although decreased susceptibility to these antimicrobials has resulted in few remaining effective treatments for enteric fever [12, 13].

Sustainable infrastructural changes to tackle the root causes of enteric fever—unclean water and inadequate sanitation—remain out of reach for the majority of the population in most endemic countries. Therefore, vaccination has been championed as an effective control strategy for enteric fever [14]. However, this strategy has been hindered by the moderate efficacy of currently licensed vaccines for *S*. Typhi, which cannot be used in children <2 years old, and by the absence of a vaccine for the *S*. Paratyphi serovars. New vaccines, such as the Tybar typhoid conjugate vaccine (TCV), recently licensed in India and prequalified by the World Health Organization (WHO), are more efficacious and immunogenic in infants [15, 16]. The WHO’s Strategic Advisory Group of Experts recently recommended the introduction of TCVs to help control the burden of enteric fever, with priority given to countries with the highest burden of disease or high AMR [17].

Implementation of these novel vaccines requires a precise understanding of the burden—and, in particular, the mortality rate—associated with enteric fever. A recent analysis showed that between 86% and 98% of the disability-adjusted life-years caused by typhoid fever were attributed to death in 5 settings in India, Kenya, and Vietnam [18]. Moreover, uncertainty in the case fatality rate (CFR) was among the factors responsible for the largest proportion of uncertainty in the cost-effectiveness of TCV delivery strategies [18]. These findings underline the importance of a better understanding of enteric fever mortality. Therefore, we conducted a systematic review and meta-analysis to aggregate all available evidence on enteric fever mortality. We aimed to estimate the CFR, quantify the uncertainty, and explore the impact of potential prognostic factors, such as age and the presence of AMR.

## METHODS

The reporting of this systematic review and meta-analysis adhere to the PRISMA (Preferred Reporting Items for Systematic Reviews and Meta-Analyses) guidelines and an a priori specified protocol registered in PROSPERO (registration No. CRD42017057428) [19, 20].

### Study Eligibility and Selection

Eligible studies were identified according to predefined inclusion and exclusion criteria (Table 1). We searched MEDLINE, PubMed Central, Embase, and Web of Science for eligible articles using terms related to “mortality,” “typhoid fever,” and “paratyphoid fever” on 11 January 2017, without language restrictions. A detailed overview of the search strategy is provided in the Supplementary Material (Supplement 1): Supplementary Figures 1–3. Titles and abstracts were screened independently (Z. P. and N. J. S.), and eligibility was confirmed in a full-text screening. Articles were excluded if the study was limited to a subset of the population (eg, children) (Table 1). We identified additional eligible articles by screening the reference list of included articles. Screening of articles in foreign languages was carried out in duplicate. Any discrepancies were resolved through consensus or discussion with a third reviewer (J. B. or V. E. P.).

**Table 1.** Inclusion and Exclusion Criteria

Inclusion criteria
The study is an epidemiological study of any design.
The study is an intervention study, but the estimates will be based only on the nonintervention group or the group administered the “gold standard” intervention.
The study assesses mortality rate associated with infection by *S*. Typhi or *S*. Paratyphi.
The study population under investigation is representative/typical for the country demography; ie, the study population age distribution covers all ages.
The study confirms *S*. Typhi and *S*. Paratyphi based on microbial culture and/or serological test results.
The study is conducted in an endemic country.
Exclusion criteria
The study was published before 1970.
The study contains data obtained before 1970 where no antibiotics were used.
The study is not conducted in humans.
The study is a review or non–peer-reviewed publication, such as a conference abstract, letter, editorial, or report.
The study is a microbiological study, except if clinical data are presented.
The data set is described multiple times, in which case only the most recent article is included.
The study population consists of a specific subset of patients, such as HIV-positive individuals, children, adults, and travelers.

Abbreviations: HIV, human immunodeficiency virus; *S*. Paratyphi and *S*. Typhi, *Salmonella enterica* serovars Paratyphi and Typhi.

### Data Extraction

To ensure that all relevant data were extracted, we (Z. P., N. J. S., and M. A.) piloted a data extraction form on 10 randomly selected articles. The final form (Supplement 2) collected data on study characteristics, study population characteristics, diagnosis of disease, *Salmonella* serovar (if reported), CFR estimate, prognostic factors related to the CFR, AMR, and factors that may introduce bias in the CFR. Data extraction was completed independently by Z. P. and N. J. S.; foreign-language articles were extracted by the respective reviewers. Any discrepancies were resolved through consensus or discussion with a third reviewer (J. B. or V. E. P.).

### Risk of Bias Assessment

We evaluated the risk of bias according to 4 domains specified by the Cochrane risk of bias tool [21]. We evaluated selection bias (study population and type of surveillance), measurement bias (diagnostics for enteric fever), attrition bias (dropouts), and other factors that might introduce bias (Supplement 3 and Supplementary Table 1). We judged the potential sources of bias as low, unclear, or high risk of bias for each study. Moreover, we investigated publication bias by constructing a funnel plot.

### Statistical Methods

For each study, we obtained the CFR by dividing the number of patients with laboratory-confirmed enteric fever who died (*Y*) by the total number with laboratory-confirmed enteric fever (*n*). We combined information across studies using a random intercept logistic regression model [22]. We investigated heterogeneity in the estimated overall CFR using the *I*^2^ statistic [23]. As a secondary analysis, we conducted subgroup analyses, according to WHO region [24], World Bank income category [25], detection method used, *Salmonella* serovar, and presence of human immunodeficiency virus (HIV)–infected individuals, to identify possible sources of heterogeneity. Furthermore, we assessed a possible association between age or AMR and the CFR among studies with sufficient data. Finally, we evaluated the sensitivity of the overall estimate to data from individual studies by performing a leave-one-out validation. All statistical analyses were conducted at a 5% significance level using the statistical software package “meta” in R (version 3.3.3) [26].

## RESULTS

**Figure 1.**
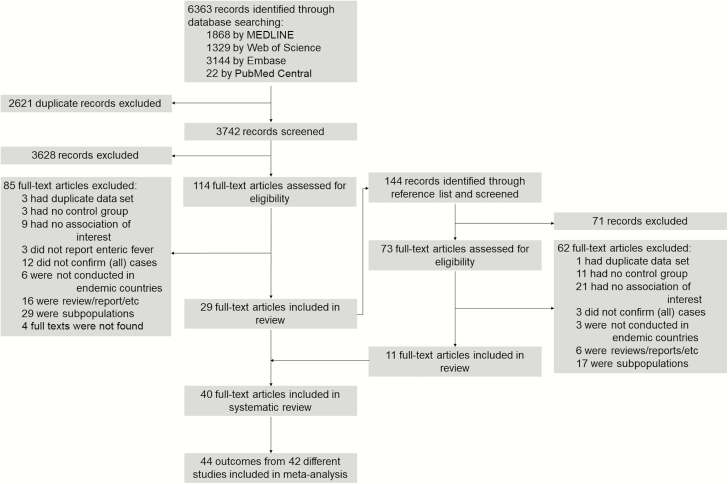
Flow diagram of the study selection process.

Of the 6363 records identified, we screened 3742 titles and abstracts after deduplication, and conducted a full-text review for 114 articles (Figure 1). Twenty-nine articles met our inclusion criteria. Eleven additional articles were identified through cross-checking of references, resulting in a total of 40 articles being included in the systematic review and meta-analysis (Figure 1). Seven articles were written in French [27–33] and 1 in Spanish [34]; the remaining articles were in English. Of the 40 articles included, 2 (Butler and colleagues [35] and Van Den Bergh and colleagues [36]), each reported mortality rates for enteric fever in 2 independent studies [35, 36]. Two other articles (Rao and colleagues [37] and Maskey and colleagues [38]) provided separate numbers of deaths for *S*. Typhi and *S*. Paratyphi. Therefore, 44 outcomes from 42 distinct studies were included in the meta-analysis.

Table 2 describes the characteristics of the included studies. Seventeen studies (40.5%) were conducted in Africa, 22 (52.4%) in Asia, and 3 (7.1%) in North America. The majority of the studies in Asia were conducted in India (7 of 22), Vietnam (4 of 22), or Indonesia (3 of 22). Among African countries, Nigeria (4 of 17) and Senegal (3 of 17) were the most represented. Because we did not exclude articles based on epidemiological design, 2 randomized controlled trials were included. All the remaining studies were observational studies, and 21 studies included hospitalized patients only. The methods to detect *S*. Typhi and *S*. Paratyphi varied among the 42 studies; 23 studies (54.8%) used a combination of a serological test and microbiological cultures to detect the organisms, and 18 (42.9%) used culture of blood or other specimens only. Fourteen studies (33.3%) included individuals infected with either *S*. Typhi or *S*. Paratyphi, but only 2 studies provided separate mortality estimates for *S*. Typhi and *S*. Paratyphi, and 28 studies (66.7%) included only *S*. Typhi in their analysis.

**Table 2.** Characteristics of Included Studies^a^

Authors	Location	Study Type	Duration	Detection Method	*Salmonella* Serovar	Deaths, No.^b^	Patients, Total No.^c^	Comments
Seydi [A1]	Dakar, Senegal	R	1996–2003	Culture: blood	*S.* Typhi*, S.* Paratyphi	8	36	Some patients admitted multiple times
Tohme [A2]	Beirut, Lebanon	R	1995–2002	Culture: blood, urine Serology: Widal test	*S.* Typhi*, S.* Paratyphi, not specified	1	70	Death occurred in 1 patient with acute respiratory distress syndrome and concurrent cytomegalovirus; *Salmonella* type was unspecified in 1 patient
Reynaud [A3]	Rabat, Morocco	R	1966–1970	Culture: blood; serology: not specified	*S.* Typhi*, S.* Paratyphi	15	434	
Papa [A4]	NA, Algeria	CS	1965–1970	Culture: blood; serology: Widal test	*S.* Typhi*, S.* Paratyphi	11	337	
Gallais [A5]	Abidjan, Ivory Coast	CS	1976–1980	Culture: blood, stool; serology: Widal test; other: not specified	*S.* Typhi	8	213	
Derrien [A6]	Dakar	CS	1977	Culture: blood, bile, stool;serology: Widal test	*S.* Typhi*, S.* Paratyphi	3	55	2 Deaths in patients with co-infection
Lefebvre [A7]	Dakar	R	1995–2002	Culture: blood, stool; serology: Widal test	*S.* Typhi	1	70	
Mendoza-Hernandez [A8]	Mexico City, Mexico	R	1972	Culture: blood, bone marrow,urine, stool, rose spot	*S.* Typhi	60	1676	
Butler [A9]	Jakarta, Indonesia	RCT	1976–1977	Culture: blood, stool; serology: not specified	*S.* Typhi	2	27	Included only patients treated with chloramphenicol
Butler [A9]	Saigon, Vietnam	RCT	1975	Culture: blood; serology: not specified	*S.* Typhi	1	11	Included only patients treated with ampicillin
Van Den Bergh [A10]	Yogyakarta, Indonesia	R	1952–1956	Culture: blood, bone marrow	*S.* Typhi	6	61	
Van Den Bergh [A10]	Semarang, Indonesia	CS	1989–1990	Culture: blood, bone marrow	*S.* Typhi	5	105	
Rao [A11]	Manipal, India	CS	1990–1991	Culture: blood; serology: Widal test	*S.* Typhi	1	102	Serological test result confirmed by culture
					*S.* Paratyphi	0	27	
Maskey [A12]	Kathmandu, Nepal	CS	2004–2004	Culture: blood	*S.* Typhi	0	408	
					*S.* Paratyphi	0	200	
Abucejo [A13]	Tagbilaran, Philippines	CS	1994–1997	Culture: blood; serology: not specified	*S.* Typhi	9	422	Serological test result confirmed by culture
Brown [A14]	Mentekab, Malaysia	CS	1975–1979	Serology: Widal test	*S.* Typhi*, S.* Paratyphi	2	121	Some patients admitted multiple times
Hoa [A15]	Ho Chi Minh City, Vietnam	CS	1993–1994	Culture: blood	*S.* Typhi*, S.* Paratyphi	1	302	
Kabir [A16]	Rajshahi, Bangladesh	CS	2000–2001	Culture: blood; serology: Widal test	Not specified	2	65	*Salmonella* serovar not mentioned
Khosla [A17]	Rohtak, India	PC	1991–1992	Culture: blood, bone marrow;serology: Widal test	*S.* Typhi	12	180	Serological test result confirmed by culture
Koh [A18]	Singapore (nationwide)	S	1970–1974	Culture: blood, urine, stool;serology: not specified	*S.* Typhi*, S.* Paratyphi	20	1004	
Lin [A19]	Dong Thap province, Vietnam	S	1995–1996	Culture: blood	*S.* Typhi	0	56	
Mathur [A20]	Jaipur, India	R	1960–1969	Culture: blood Serology: not specified	Not specified	247	2284	*Salmonella* serovar not mentioned
Mukherjee [A21]	Calcutta, India	CS	1989–1990	Culture: blood, stool Serology: Widal test	*S.* Typhi	6	46	Serological test result confirmed by culture
Parande [A22]	Solapur, India	CS	NA	Culture: blood; serology: Widal test	*S.* Typhi*, S.* Paratyphi	3	172	
Parry [A23]	Ho Chi Minh City and Cao Lanh, Vietnam	CS	1993–1999	Culture: blood, bone marrow	*S.* Typhi	3	581	
Phetsouvanh [A24]	Vientiane, Laos	CS	2000–2004	Culture: blood	*S.* Typhi	3	218	
Sen [A25]	Burla, India	NA	NA	Culture: blood, urine; serology: Widal test	*S*. Typhi, *S*. Paratyphi	1	54	Only *S*. Typhi could be isolated in 5 cases, but we assume that *S*. Paratyphi could also be present
Shahunja [A26]	Dhaka, Bangladesh	CC	2009–2013	Culture: blood, stool	*S.* Typhi	0	60	
Walia [A27]	New Delhi, India	R	2001–2003	Culture: blood; serology: not specified	*S.* Typhi*, S.* Paratyphi	4	88	
Abdurrahman [A28]	Kaduna, Nigeria	R	1973–1974	Culture: blood, stool, urine; other: intestinal lesions	*S.* Typhi*, S.* Paratyphi	18	150	
Abraham [A29]	Addis Ababa, Etiopia	R	1975–1980	Culture: blood	*S.* Typhi	6	50	
Akinyemi [A30]	Lagos State, Nigeria	R	1999–2008	Culture: blood, bone marrow,stool, urine; serology: Widal test	*S.* Typhi	227	30210	Serological test result was confirmed by culture
Ameh [A31]	Sokoto, Nigeria	R	1985–1989	Culture: blood, stool, urine	*S.* Typhi	8	531	We assume number of deaths was known only for inpatients
Breiman [A32]	Nairobi, Kenya	S	2007–2009	Culture: blood	*S.* Typhi	0	135	Only data from Kibera site are included
Elegbeleye [A33]	Lagos, Nigeria	R	1966–1970	Culture: blood serology: not specified	*S.* Typhi	12	52	
Feasey [A34]	Blantyre, Malawi	R	2011–2013	Culture: blood	*S.* Typhi	10	403	
Keddy [A35]	South Africa (nationwide)	S	2003–2013	Culture: blood, cerebrospinalfluid, other body sites	*S.* Typhi	16	237	
Popkiss [A36]	Cape Town, South Africa	CC	1978	Culture: blood, stool, urine;serology: Widal test	*S.* Typhi	0	61	
Weeramanthri [A37]	Fajara, Gambia	R	1981–1986	Culture: blood	*S.* Typhi	3	74	
Wicks [A38]	Harare, Zimbabwe	R	1966–1969	Culture: blood, stool, urine;serology: Widal test; other: intestinal lesions	*S.* Typhi	17	243	
Grell [A39]	Roseau, Dominica	CS	1972–1976	Culture: blood, stool, urine;serology: not specified	*S.* Typhi	1	78	Serological test result was confirmed by culture
Macfarlane [A40]	Kingston, Jamaica	R	1982–1983	Culture: blood	*S.* Typhi	0	14	

Abbreviations: CC, case-control study; CS, cross-sectional study; NA, not available; PC, prospective cohort study; R, retrospective study based on patient files; RCT, randomized control trial; S, surveillance; *S*. Paratyphi and *S*. Typhi, *Salmonella enterica* serovars Paratyphi and Typhi.

^a^The full citations for the included articles are provided in Supplement 4.

^b^Deaths among patients with laboratory-confirmed enteric fever.

^c^Total number of patients with laboratory-confirmed enteric fever.

**Figure 2.**
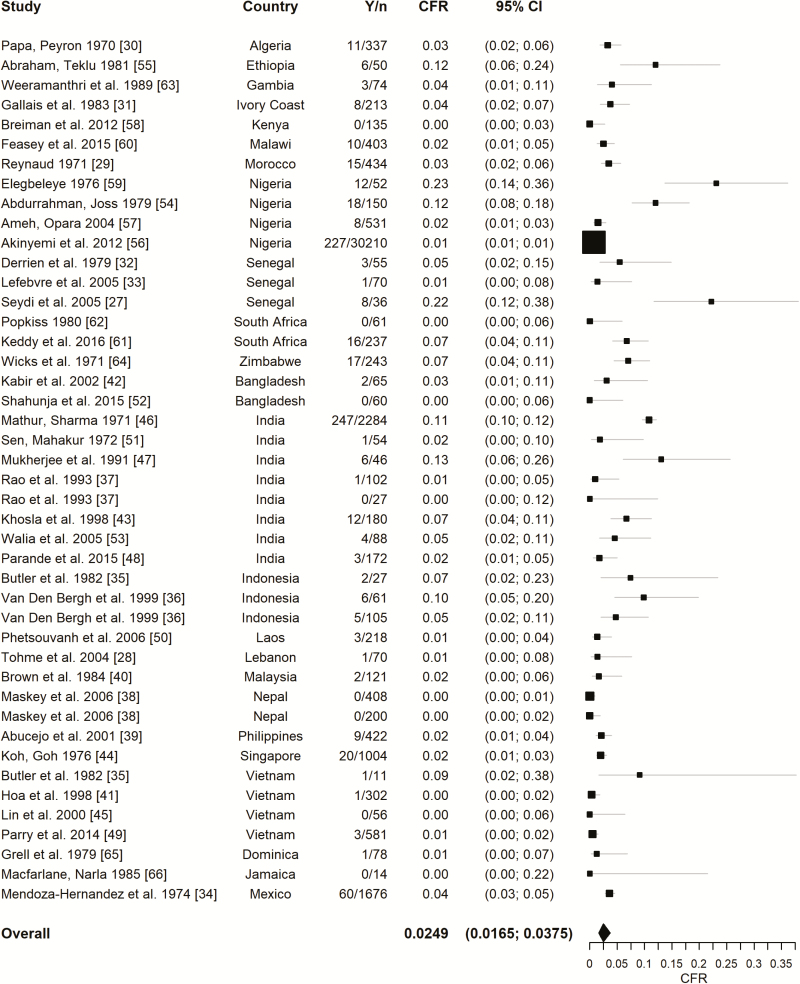
Forest plot for the case fatality rate (CFR) of enteric fever. The overall estimate was obtained from a random intercept logistic regression model. *I*^2^ = 94.7%. The 95% confidence intervals (CIs) of the individual studies were Wilson score intervals, and the CI of the overall estimate was based on a *t* distribution. Abbreviations: *n,* number of cases; *Y,* number of deaths.

We estimated an overall CFR of 2.49% (95% CI, 1.65%–3.75%; *I*^2^ = 94.7%). The CFR among hospitalized patients was 4.45% (95% CI, 2.85%–6.88%; *I*^2^ = 87.0%; n = 21). Study-specific CFRs ranged from 0% to 23%, with the highest rates observed in Nigeria and Senegal; the corresponding 95% prediction interval ranged between 0.25% and 26.36% (Figure 2) [27, 59]. The study conducted by Elegbeleye and colleagues [59] in Nigeria attributed the high CFR to very poor sanitary conditions. Seydi and colleagues [27] reported that 10 of 36 individuals in their study in Senegal were HIV-infected; however, they did not report deaths from enteric fever separately for HIV-infected versus uninfected individuals. Seven studies (reporting 8 outcomes) conducted in Africa, Asia, and North America, reported no deaths from enteric fever, with the number of cases varying from 14 to 406 [37, 38, 45, 50, 58, 62, 66]. Leave-one-out validation resulted in a CFR ranging from 2.37% to 2.71%, indicating that the CFR of the full set is robust to the influence of any single study (Supplement 4: Supplementary Table 2).

There was considerable heterogeneity in the CFR between the studies, with an estimated *I*^2^ of 94.7%, which we investigated further using subgroup analyses (Table 3; Supplement 5: Supplementary Figures 4–9). When studies were grouped according to World Bank income level, the lower-middle income countries had considerably less heterogeneity (*I*^2^ = 35.5%) than the low-income countries; however, the CFR did not differ significantly among the different income levels. In addition, we grouped studies according to WHO region and observed that there was lower heterogeneity in the Western Pacific region (*I*^2^ = 40.7%), which also had the lowest CFR (1.26%; 95% CI, 0.66%–2.36%); the greatest heterogeneity was observed in the African region (*I*^2^ = 94.2%), which also had the highest CFR (3.82%; 95% CI, 1.97%–7.26%). We also observed substantial heterogeneity for studies conducted in the same country, as well as within the same setting (Supplement 5: Supplementary Figure 9). Other potential factors were investigated, such as the *Salmonella* serovar, presence of HIV-infected individuals, and the detection method used to identify the organism; however, none of these could explain the heterogeneity in the CFR (Table 3; see Supplement 5 for details).

**Figure 3.**
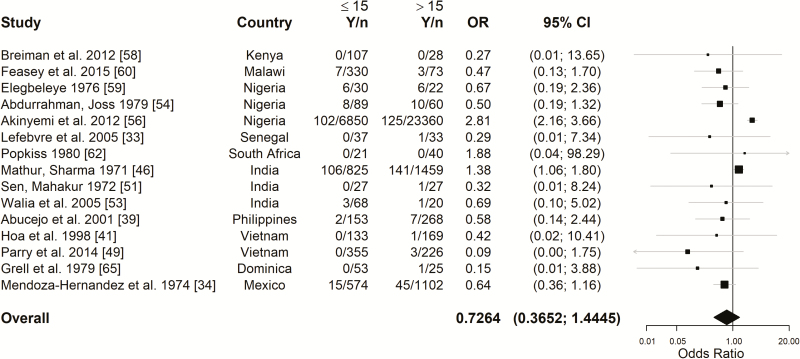
Forest plot for the odds of dying of enteric fever in children (≤15 years of age) versus adults (>15 years of age). The overall estimate was obtained from a random intercept logistic regression model. *I*^2^ = 76.3%. The 95% confidence intervals (CIs) of the individual studies were Wilson score intervals, and the 95% CI of the overall estimate was based on a *t* distribution. Abbreviations: *n,* number of cases; OR, odds ratio; *Y,* number of deaths.

Next, we assessed the potential impact of age on mortality rates for enteric fever by comparing the odds of death from enteric fever in children (≤15 years of age) versus adults (>15 years of age), which was reported in 15 studies (Figure 3; see Supplement 6: Supplementary Table 3 for details and assumptions). The estimated overall odds ratio (OR) comparing children with adult (>15 years of age) was 0.73 (95% CI, .37–1.44). All studies except 2 showed no significant difference between children and adults; the 2 studies with a statistically significant effect showed that the odds of dying of enteric fever were higher in children than in adults [46, 56].

**Table 3.** CFR According to Stratification Factors^a^

Categories	Studies, No.	*I* ^2^, %	CFR (95% CI), %
World Bank income level			
Low	29	96.72	2.28 (1.19–4.31)
Lower-middle	12	35.50	3.17 (2.29–4.38)
Upper-middle	2	6.66	5.21 (0–99.84)
WHO region			
African	16	94.20	3.82 (1.97–7.26)
Eastern Mediterranean	2	0	3.17 (.13–45.28)
Americas	3	0	3.45 (2.00–5.89)
South-East Asia	15	90.92	2.27 (.91–5.55)
Western Pacific	8	40.72	1.26 (.66–2.36)
Detection method			
Serology and cultures	24	92.96	3.22 (2.08–4.97)
Cultures only	19	94.48	1.63 (.63–4.12)
Inclusion of HIV-infected individuals			
Yes	5	84.91	4.20 (1.02–15.74)
No		95.03	2.32 (1.49–3.61)
Serovar			
*S*. Typhi and *S*. Paratyphi	12	85.11	3.08 (1.55–6.04)
*S*. Typhi only	28	93.26	2.36 (1.37–4.04)
Not specified	2	0	10.6 (4.82–21.74)
Countries with multiple estimates			
Bangladesh	2	0	1.60 (0–99.29)
India	7	82.57	4.52 (1.91–10.32)
Indonesia	3	0	6.74 (2.06–19.90)
Nigeria	4	97.81	4.28 (.40–33.34)
Senegal	3	75.45	6.20 (.29–60.37)
South Africa	2	62.41	2.16 (0–100)
Vietnam	4	0	0.53 (.13–2.16)

Abbreviations: CI, confidence interval; CFR, case fatality rate; HIV, human immunodeficiency virus; *S*. Paratyphi and *S*. Typhi, *Salmonella enterica* serovars Paratyphi and Typhi; WHO, World Health Organization.

^a^More information is provided in Supplement 5.

**Figure 4.**
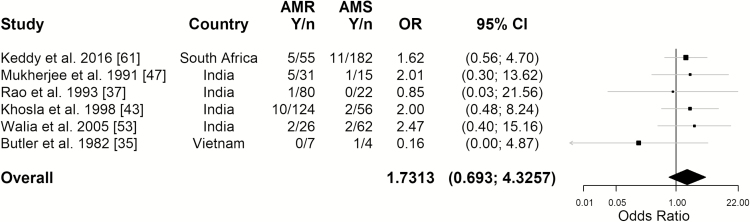
Forest plot for the odds of dying of enteric fever when infected with a resistant versus a sensitive strain. The overall estimate was obtained from a random intercept logistic regression model. *I*^2^ = 0%. The 95% confidence intervals (CIs) of the individual studies were Wilson score intervals, and the 95% CI of the overall estimate was based on a *t* distribution. Abbreviations: AMR, antimicrobial resistance; AMS, antimicrobial sensitivity; *n,* number of cases; OR, odds ratio; *Y,* number of deaths.

Six studies reported information on deaths separately for antimicrobial-resistant versus antimicrobial-sensitive strains (Figure 4; see Supplement 6 and Supplementary Table 4 for details and assumptions). The estimated probability of dying of enteric fever when infected with an AMR strain was 6.84% (95% CI, 3.17%–14.17%; *I*^2^ = 38.0%). We found that the OR associated with death from infection with a resistant versus a susceptible strain was 1.7 (95% CI, .69–4.33) (Figure 4) [35, 37, 43, 47, 53, 61]; thus, the difference in CFR between the 2 groups was not statistically significant.

**Figure 5.**
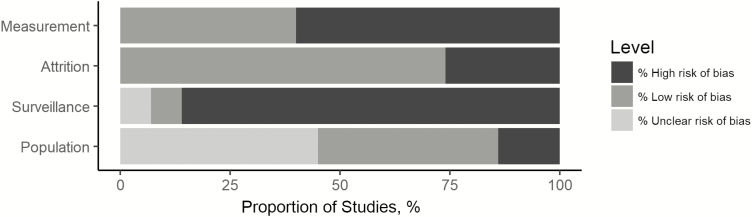
Risk of bias assessment. The proportion of all studies (n = 42) judged to have high (*black*), low (*dark gray*), or unclear (*light gray*) risk of bias is plotted for each of the 4 categories: measurement, attrition, surveillance, and population bias. Studies reporting a separate case fatality rate for typhoid and paratyphoid fever were included only once.

Figure 5 displays the results of the risk of bias assessment, summarizing the proportion of outcomes judged as high, low, or unclear risk of bias. Results of the bias assessment for each individual study are provided in Supplementary Table 5 (Supplement 7). The main source of risk of bias was the type of surveillance. Only 3 studies (7.1%) performed active surveillance to identify enteric fever cases. More than 50% of the studies did not use the gold standard diagnostic (blood or bone marrow culture) to identify cases. Often, it was not possible to determine the risk of bias attributable to the study population because of limited information. We judged 31 studies (73.8%) to have low attrition bias owing to the lack of dropouts. In addition, the funnel plot showed an asymmetric distribution of point estimates, yielding evidence of publication bias, but this was driven by 1 large study (Supplement 8: Supplementary Figure 10) [56].

## DISCUSSION

We estimated an overall CFR of 2.49% (95% CI, 1.68%–3.89%) from the existing literature, and a CFR among hospitalized patients of 4.45% (95% CI, 2.85%–6.88%). The probability of death from enteric fever did not differ significantly between children and adults (OR, 0.73; 95% CI, .37–1.44) or between AMR and sensitive strains (1.7; 95% CI, .69–4.33), which can mean either that there is no difference or that we did not have enough evidence to detect one. Nevertheless, our results highlight the potential severity of AMR infections, for which we estimated the CFR was 6.84% (95% CI, 3.17%–14.17%).

We found considerable heterogeneity in published estimates of the CFR for enteric fever. This heterogeneity persisted even when performing subgroup analyses of our data by WHO region, World Bank income level, detection method, and *Salmonella* serovar (Table 3; Supplement 5). Most likely, heterogeneity cannot be explained solely by a single factor. Key differences between the studies will depend on local management of the disease, local policies, differences in culture and access to care, and so forth. Differences in reporting (and lack of reporting) did not allow us to explore more factors. Estimates can vary considerably, even within countries (Table 3; Supplement 5). Given the amount of heterogeneity detected in the meta-analysis, the results should be interpreted with caution.

Until recently, a CFR of 1% has been assumed in estimating the mortality burden of enteric fever [6–8]. Crump and colleagues [7] based this on conservative estimates from hospital-based studies, and others have followed suit in the absence of new data. Mogasale and colleagues [6] built on a systematic literature review conducted by Crump and colleagues [67] and estimated a case-weighted mean CFR of 2.8% (95% CI, 2.0%–3.6%) in hospitalized patients. The results from their meta-analyses were used to conduct a bootstrap analysis as an alternative scenario in estimating the mortality burden of typhoid fever; however, the details of the meta-analysis are not provided [6]. Lozano and colleagues [68] used surveillance data from the Brazilian Ministry of Health’s Information System for Notifiable Diseases and reported a mean CFR of 0.996%. Although systematic literature searches have been conducted, the authors summarized the CFR by simply aggregating the deaths and cases by certain factors or by providing the median and range, rather than conducting a formal meta-analysis [67, 69].

Reliable estimates of the CFR of typhoid fever, its uncertainty, and how it varies depending on predictors such as age of patients, geographic region, and prevalence of AMR are essential for evaluating the cost-effectiveness of TCV delivery strategies. A recent analysis highlighted the fact that life-years lost due to death accounted for the vast majority of disability-adjusted life-years attributable to typhoid fever, and this was an important factor in determining the optimal vaccination strategy [18]. Thus, our revised estimate of the CFR for enteric fever, and its associated uncertainty, should be incorporated in future models of typhoid burden and cost-effectiveness of interventions.

The estimate found in this study was mostly derived from hospitalized cases and from passive surveillance studies. This might introduce bias in the CFR, because passive surveillance will most likely underestimate the number of cases [70]. The question then is whether the cases that were not detected are more or less ill. Saha and colleagues [71] argued that passive surveillance is biased toward more severe illness, because only very ill patients seek care. Following this reasoning, passive surveillance could overestimate the CFR for enteric fever. On the other hand, the CFR may be higher in the absence of treatment [36, 72]. An estimate of the CFR derived solely from active surveillance studies may be optimal, because all cases are included and followed up. However, implementation of active surveillance for febrile illness is difficult because countries where the disease is endemic do not have sufficient resources [73].

Only 3 studies identified in our search conducted active surveillance, and among these studies, the CFR was 0.28% (95% CI, 0%–99.66%) [58, 61, 62]. Active surveillance might also underestimate the CFR owing to the enhanced clinical management of febrile patients in the context of the study. Ill patients might be encouraged to seek care and receive an appropriate diagnosis earlier than they would otherwise, thereby preventing their illness from progressing, leading to underestimation of the CFR [67]. Similar reasoning can be applied to randomized control trials in which patients receive enhanced care, potentially leading to an underestimate of the CFR.

The CFR was also lower among studies that relied solely on culture confirmation of cases (1.63%; 95% CI, .63%–4.12%) than in those that used both culture and serology to detect cases (3.22%; 95% CI, 2.08%–4.97%), although the difference was not statistically significant (Supplement 5). Serological tests for enteric fever, notably the Widal test, suffer from poor sensitivity. Therefore, studies that included serologically detected cases may include cases (and deaths) with other conditions. This could lead to an overestimate of the CFR if these false-positive cases were more severe than the culture-confirmed cases. In addition, clinical features may be misleading and laboratory tests may be unreliable in the presence of intestinal perforations, resulting in an underestimation of the CFR of enteric fever [69]. The CFR has been found to be higher among patients with intestinal perforation (15.4%), but the proportion of enteric fever episodes leading to severe outcomes is not yet well described [74, 75]. Two ongoing surveillance programs in Africa and Asia will hopefully provide more insights in the near future into the proportion of cases leading to severe complications.

Our search strategy and inclusion/exclusion criteria were designed to inform our overall estimate of the CFR of enteric fever in the general population. As such, we excluded studies limited, for instance, to certain age groups. Therefore, our results on the relationship between age and AMR and the CFR of enteric fever should be interpreted with caution. Further research on these specific topics is necessary.

The CFR of enteric fever remains high in endemic countries, highlighting the severity of this preventable disease. However, there is considerable heterogeneity in estimates of the CFR, and insufficient data on prognostic factors and vulnerable subpopulations. More studies are needed to estimate heterogeneity in the CFR by age and the impact of emerging AMR.

## Supplementary Data

Supplementary materials are available at *Clinical Infectious Diseases* online. Consisting of data provided by the authors to benefit the reader, the posted materials are not copyedited and are the sole responsibility of the authors, so questions or comments should be addressed to the corresponding author.

Supplementary Appendix

## Notes

***Author contributions.*** V. E. P. and J. B. conceived the study. Z. P. wrote the protocol, assisted by N. J. S., M. A., V. E. P., and J. B., and Z.P. and M.A. performed the database search. Z. P. and N. J. S. conducted the data screening and selected articles for inclusion. Z. P. and N. J. S. extracted data from French and English articles, and M. A. extracted data from Spanish articles. Z. P. performed all analyses and wrote the first draft. V. E. P. and J. B. played a guiding role in this whole process and resolved any issues when they arose. All authors revised the draft and approved the final version for publication.

***Acknowledgments.*** We thank Polina Putrik, Anna Ivanova, Oana Petrof, Stefan Catrina, Sereina Herzog, Kim Hendrickx, Andrea Torneri, Matteo Campioli, Kayoko Shioda, Ryo Horiuchi, Ezgi Tanriver, and Özgür Koc for screening titles and abstracts in languages other than English, French, and Spanish. We are grateful to Alvaro Florez Poveda, who helped with the screening and data extraction of the Spanish-language articles. We also thank Stéphanie Blaizot, Diana Hendrickx, and Niel Hens for their suggestions during laboratory meetings, and Christopher Parry and Segun Akinyemi for providing data on their studies.

***Financial support.*** This work was supported by the Bill & Melinda Gates Foundation (grant OPP1116967), the Research Foundation–Flanders (FWO; postdoctoral fellowship to J. B.), the Belgian American Educational Foundation (www.baef.be; postdoctoral fellowship to N. J. S.), and the Antwerp Study Centre for Infectious Diseases, University of Antwerp (Z. P. and J. B.).

***Potential conflicts of interest.*** All authors: No reported conflicts of interest. The funder of the study had no role in study design, data collection, data analysis, data interpretation, or writing the report. The corresponding author had full access to all the data in the study and had final responsibility for the decision to submit for publication. All authors have submitted the ICMJE Form for Disclosure of Potential Conflicts of Interest. Conflicts that the editors consider relevant to the content of the manuscript have been disclosed.
