# Supplementary material for: Case Fatality Rate of Enteric Fever in Endemic Countries: A Systematic Review and Meta-analysis
Source: Clin Infect Dis. 2018 Mar 7;67(4):628–38. doi: 10.1093/cid/ciy190 (PMC6070077; doi:10.1093/cid/ciy190)
Supplement: Supplementary Appendix [file ciy190_suppl_supplementary_appendix.docx]

**Supplement 1: Search strategy**

The searches were performed in Embase, Medline, PubMed Central, and Web of Science. No language restriction were imposed during the search. We used free text (FT) and controlled vocabulary (SH) related to the concepts ‘mortality’, ‘typhoid fever’, and ‘paratyphoid fever’.

The search strategy and results for each database are detailed below. After de-duplication, ZP removed laboratory and animal studies, conference abstracts, letters, and reviews. Then ZP and NJS independently screened titles and abstracts of the remaining relevant articles, for which we confirmed the eligibility in a full-text screening. If the abstract was unavailable, we directly examined the full-text.

**Embase**

Searched from 1970 to January 11, 2017

A detailed overview of the search terms are provided below and in Supplementary Figure 1.

1. FT: (typhoid* or (Enteric adj2 fever*) or typhus abdominalis or Salmonella typhi or Salmonella typhosa or Salmonella enterica serovar* typhi).

2. FT: paratyph*

3. FT: death* or mortalit* or fatal* or died or dying or deceased or life threatening or lethal* or casualt* or hospitali*

4. SH, paratyphi: exp paratyphoid fever/ or exp salmonella enterica serovar paratyphi a/ or exp salmonella enterica serovar paratyphi b/ or exp salmonella enterica serovar paratyphi c/

5. SH: exp typhoid fever/ or exp salmonella enterica serovar typhi/

6. SH: exp Mortality/ or hospitalization/


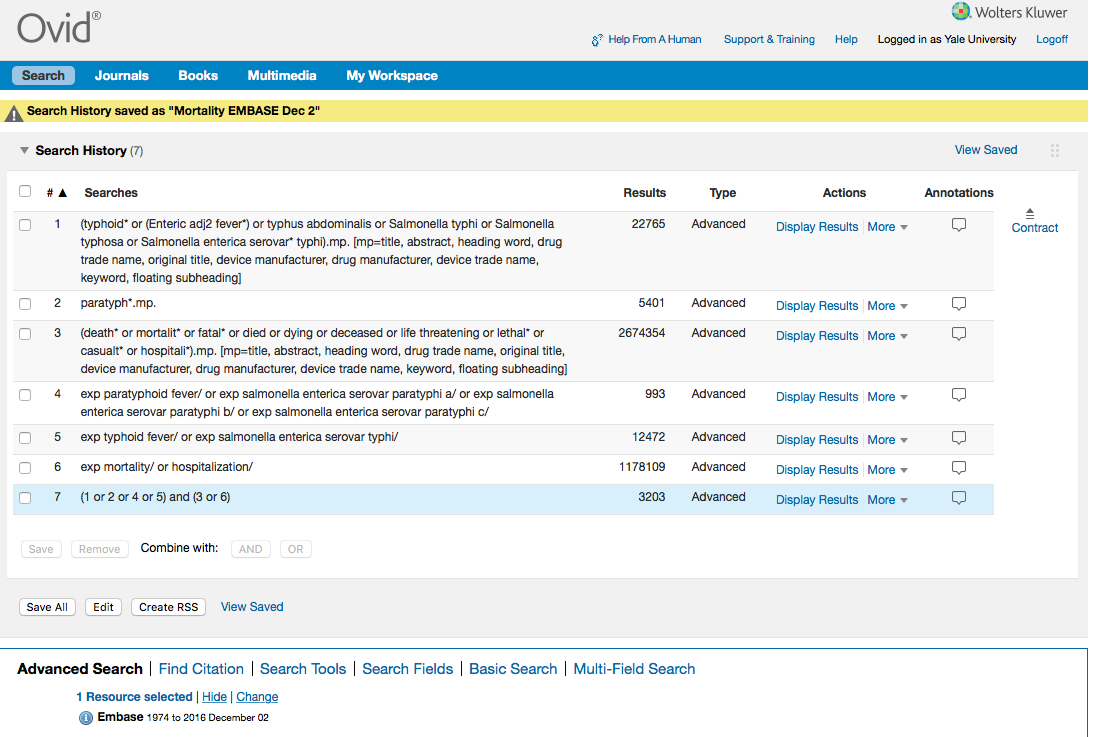


**Supplementary Figure 1: Details of the search strategy in Embase.** The screenshot dates from December 2, 2016. On January 11, 2017 the search was updated, which resulted in 3144 articles.

**MEDLINE**

Searched from 1970 to December 2, 2016

A detailed overview of the search terms are provided below and in Supplementary Figure 2.

1. FT: (typhoid* or (Enteric adj2 fever*) or typhus abdominalis or Salmonella typhi or Salmonella typhosa or Salmonella enterica serovar* typhi).

2. FT: paratyph*

3. SH: exp Salmonella typhi/ or exp typhoid fever/

4. SH: exp salmonella paratyphi a/ or exp salmonella paratyphi b/ or exp salmonella paratyphi c/ or exp Paratyphoid Fever/

5. FT: death* or mortalit* or fatal* or died or dying or deceased or life threatening or lethal* or casualt* or hospitali*

6. SH: exp Mortality/ or exp Hospitalization


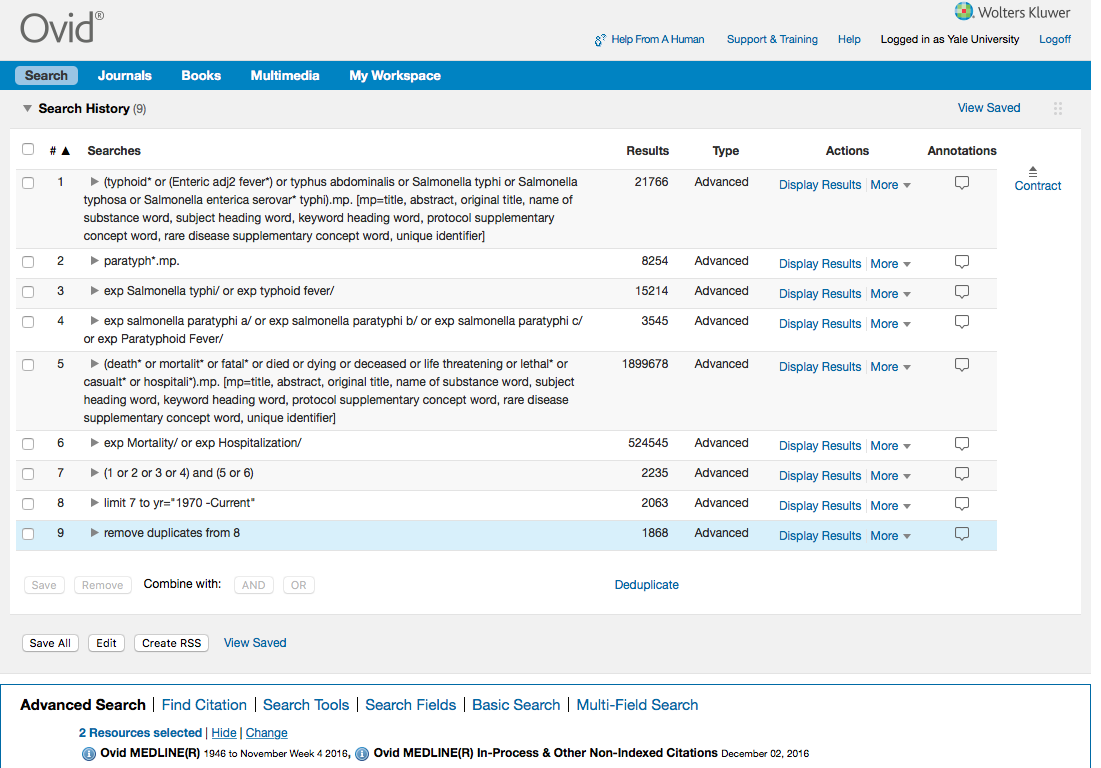


**Supplementary Figure 2: Details of the search strategy in MEDLINE**

**PubMed Central**

Searched from 1970 to December 8, 2016

A detailed overview of the search terms are provided below and in Supplementary Figure 3.

1. Typhoid* or (Enteric adj2 fever) or typhi or typhus abdominalis or abdominal typhoid

2. Paratyph*

3. Death or deaths or mortality or mortalities or Fatal or fatalities or Fatal Outcome or fatal outcomes or Case fatality or case fatalities or case fatality rate or hospitalized or hospitalised or hospitalization or hospitalisation or died or dying or deceased or life-threatening or lethal or lethality or casualty or casualties


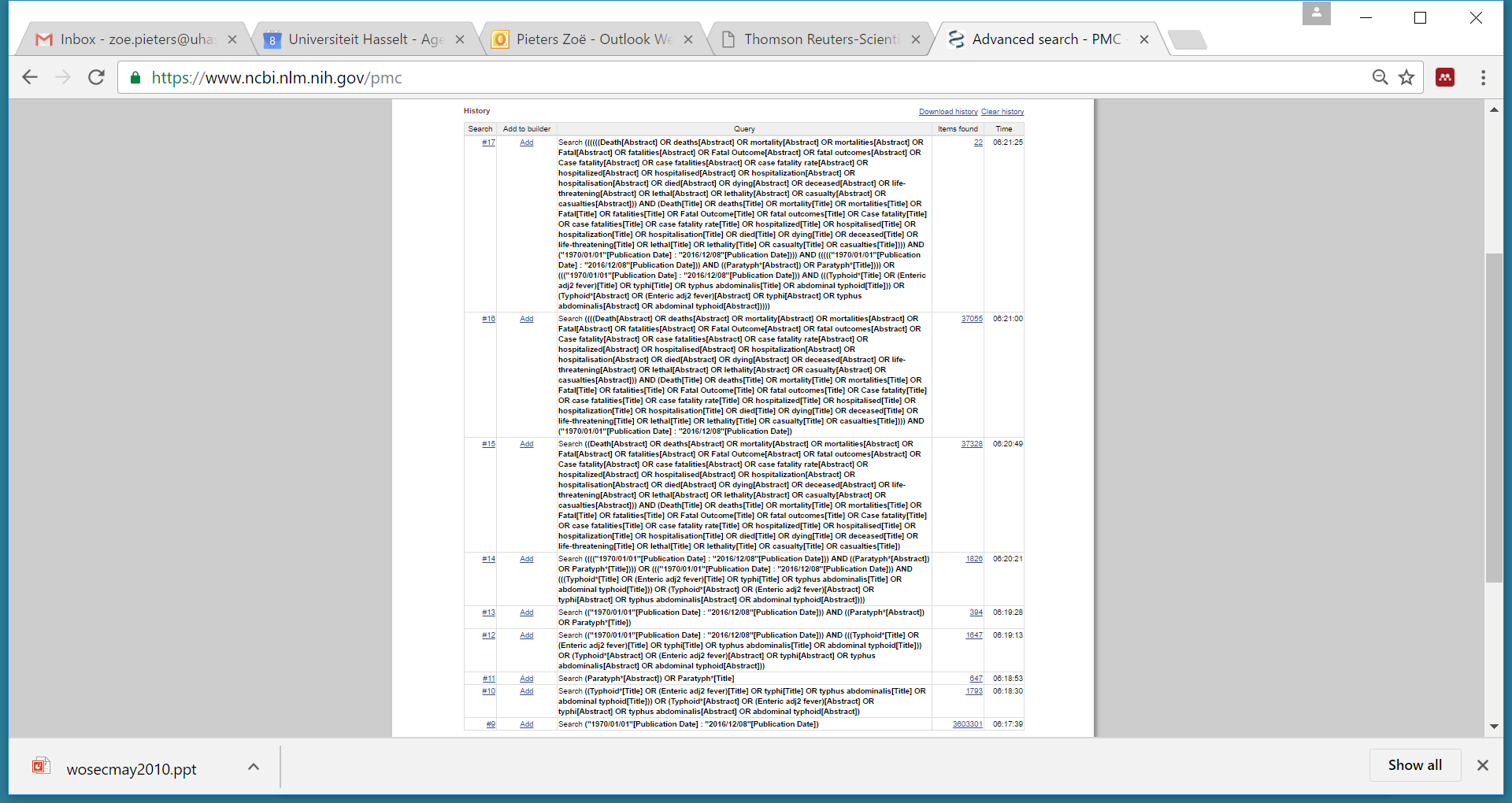


**Supplementary Figure 3: Details of the search strategy in PubMed Central**

**Supplement 2: Data extraction form**


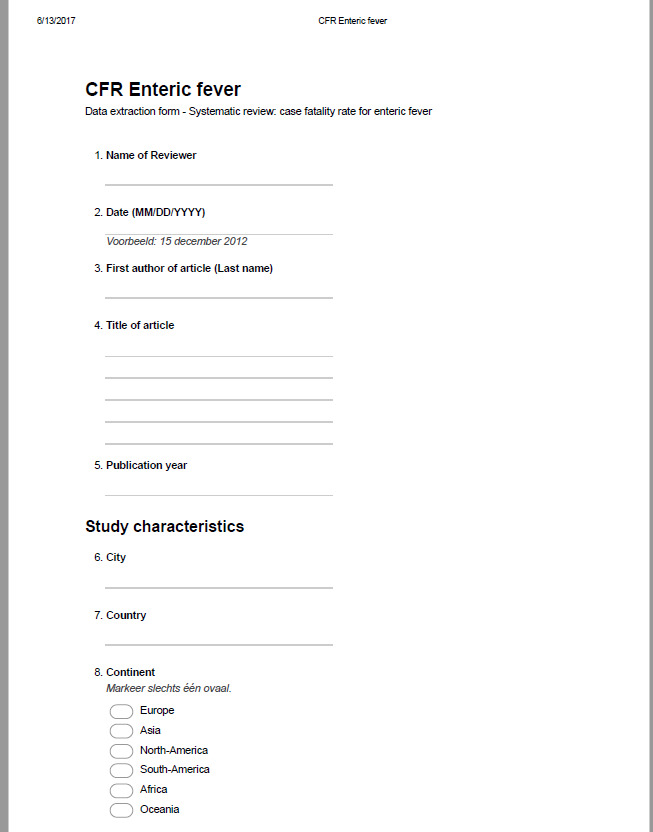


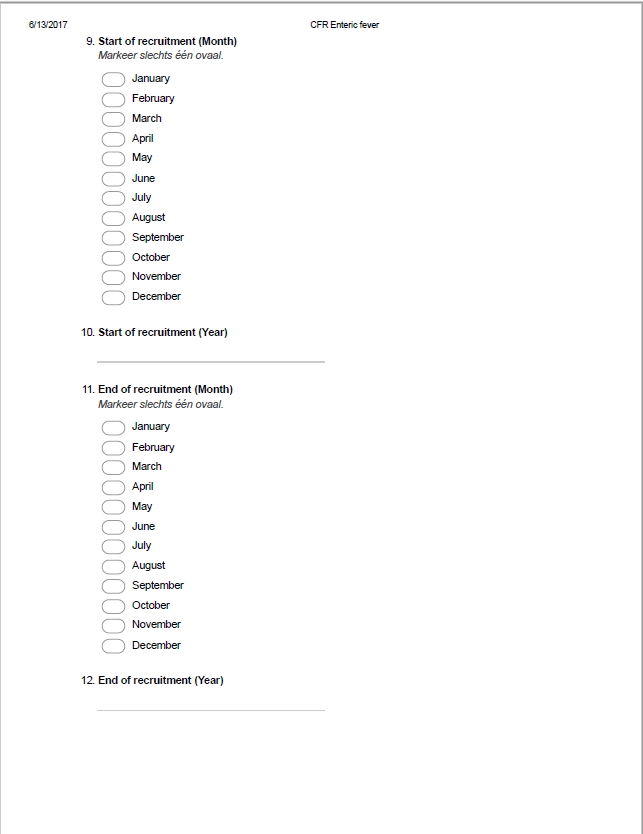


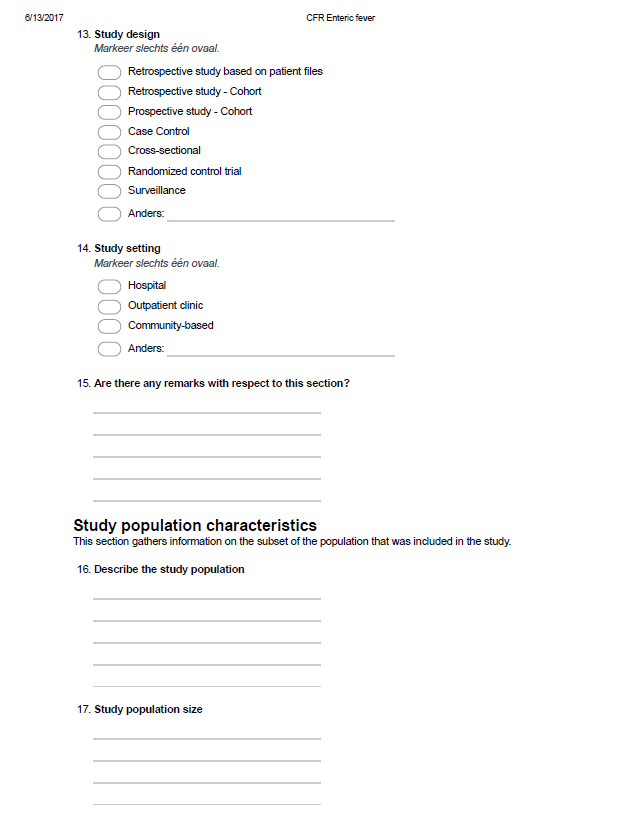


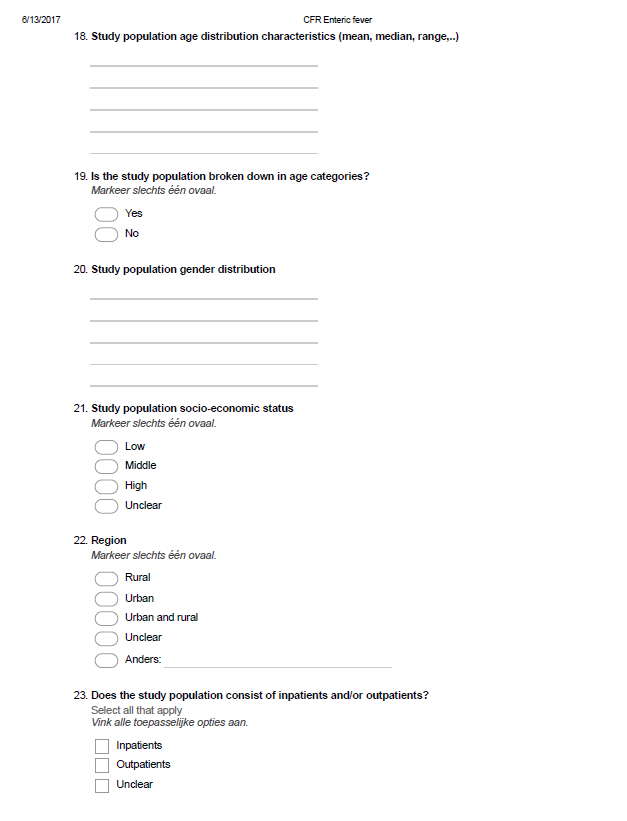


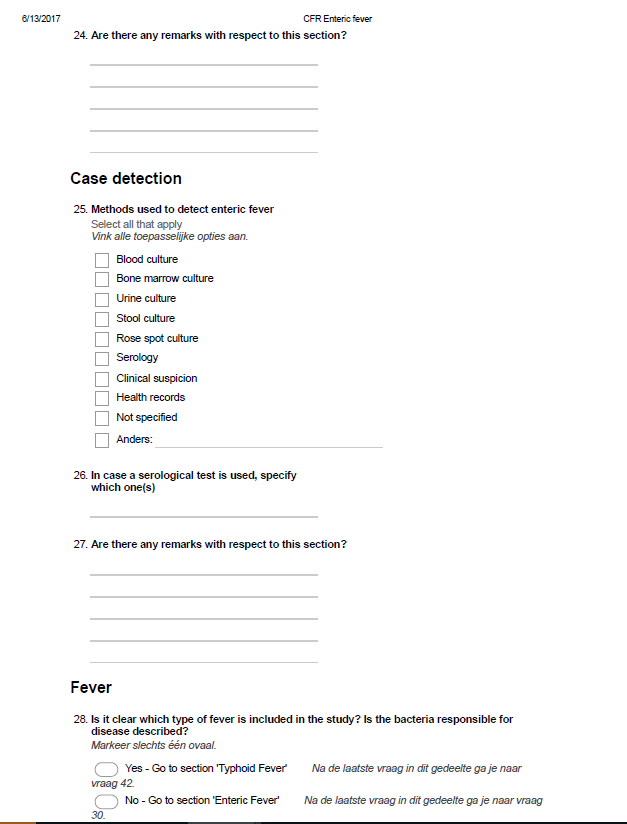


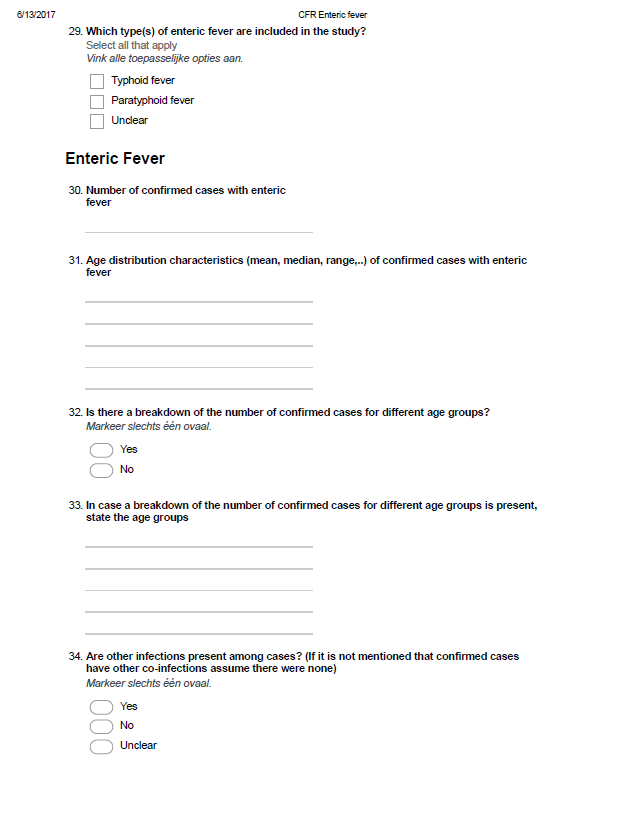


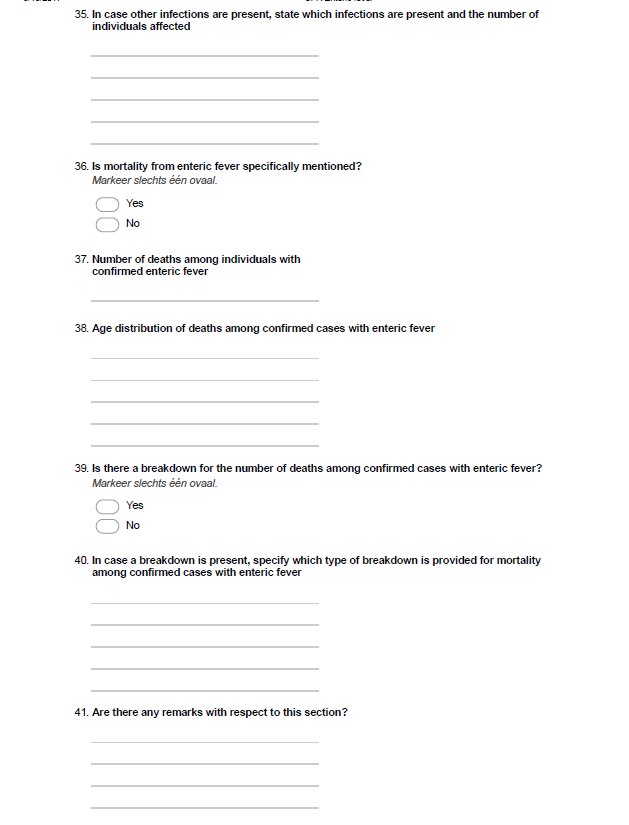


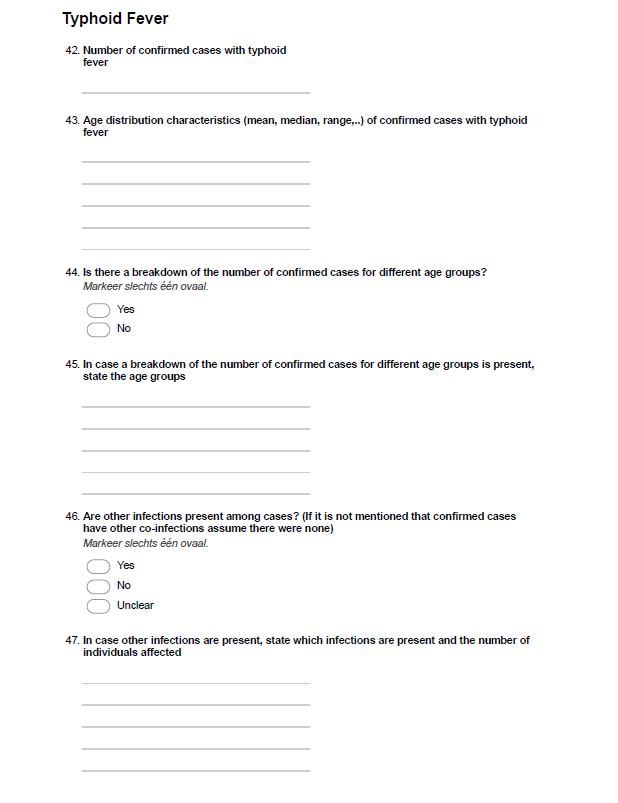


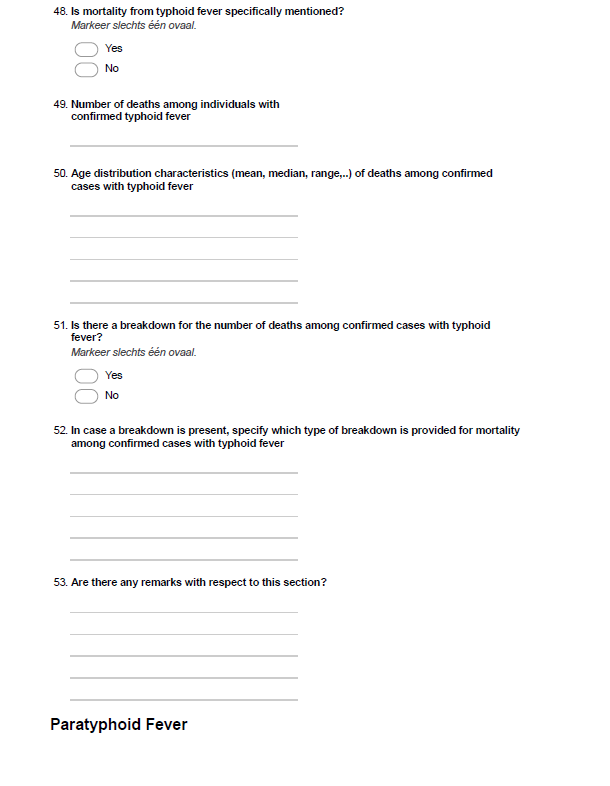


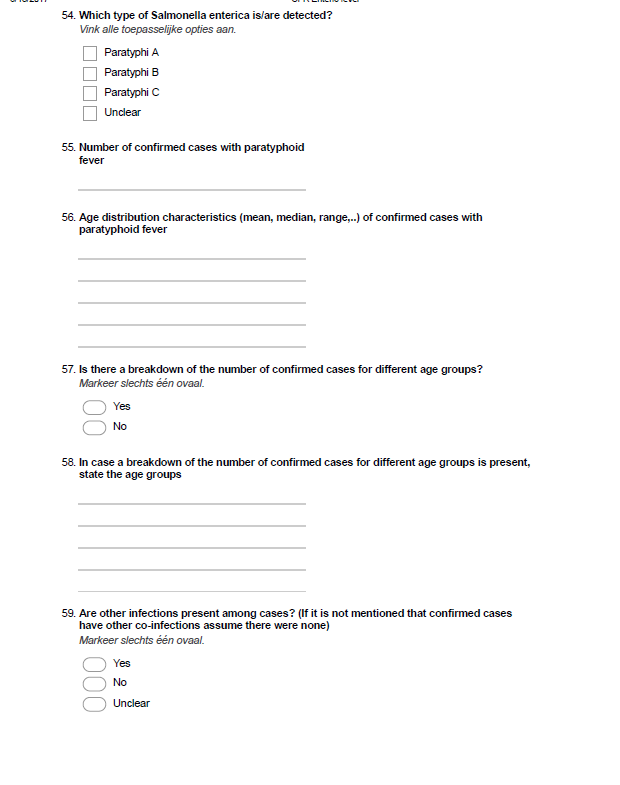


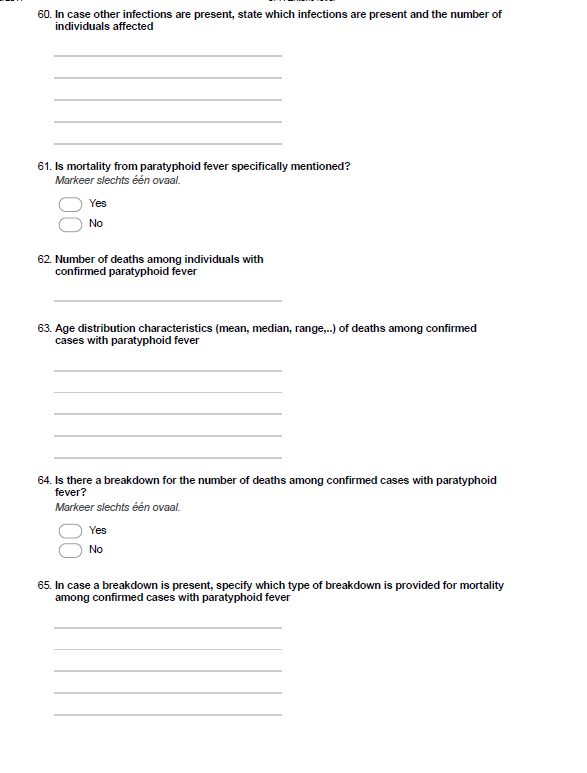

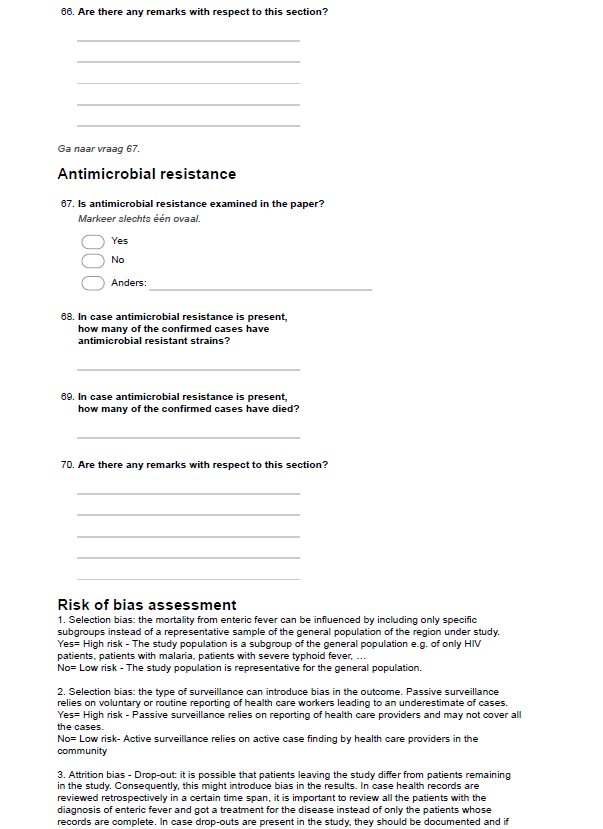


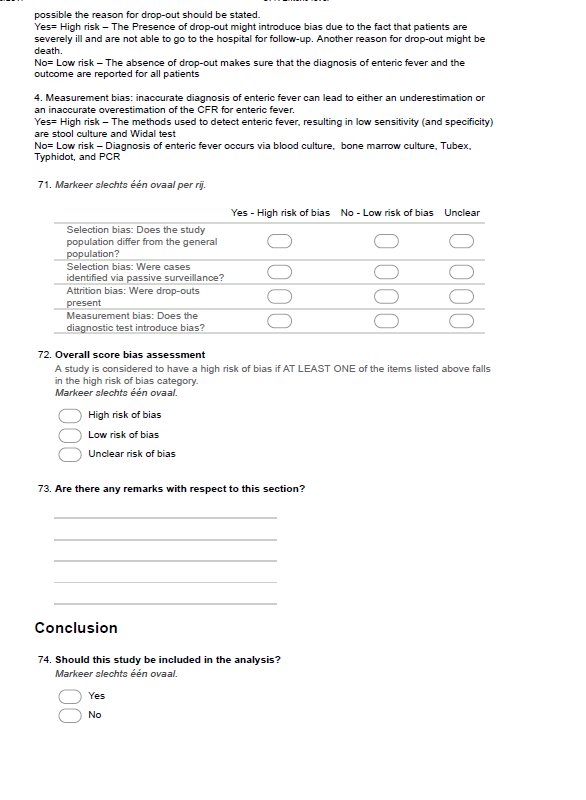


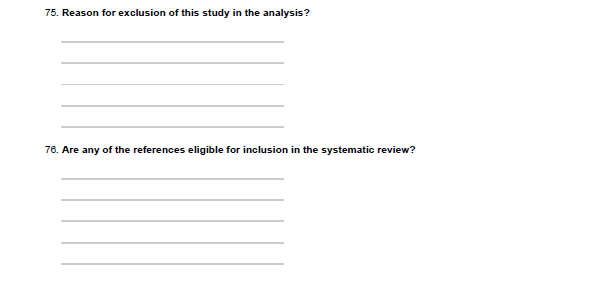


**Supplement 3: Risk of bias assessment form**

We used the four domains for risk of bias as specified by Cochrane Risk of Bias Tool to assess the potential sources for risk of bias [1]. Factors putting the outcome of interest, mortality from enteric fever, at risk of bias are listed below.

1. Selection bias – General population: the mortality from enteric fever can be influenced by including only specific subgroups instead of a representative sample of the general population of the region under study. Examples of these subgroups are patients with malaria, HIV, dengue, and pregnant women. Also, study populations consisting of only patients with severe typhoid fever are considered to be not representative of the general population.
2. Selection bias – Active and passive surveillance: the type of surveillance can introduce bias in the outcome under investigation, CFR for enteric fever. Passive surveillance relies on voluntary or routine reporting of health care workers leading to an underestimate of cases [2].
3. Attrition bias - Drop-out: it is possible that patients leaving the study differ from patients remaining in the study. Consequently, this might introduce bias in the results. In case health records are reviewed retrospectively in a certain time span, it is important to review all the patients with the diagnosis of enteric fever and got a treatment for the disease instead of only the patients whose records are complete [3,4]. In case drop-outs are present in the study, they should be documented and if possible the reason for drop-out should be stated.
4. Measurement bias: inaccurate diagnosis of enteric fever can lead to either an underestimation or an inaccurate overestimation of the CFR for enteric fever. The ‘golden’ standard for diagnosis of enteric fever, blood culture, has a low sensitivity. Also, the Widal agglutination test has a very poor sensitivity and specificity [5–7]. The diagnostic test is assigned a score related to its sensitivity and specificity to detect *S.* Typhi or *S.* Paratyphi.
5. Other: In case other concerns arise, affecting the CFR for enteric fever, that were not yet listed above can be listed here.

Supplementary Table 1 is created for evaluating the quality of each eligible study. An explanation is provided for each answer to ensure that a uniform evaluation is performed by each reviewer.

**Supplementary Table 1: Risk of bias assessment table**

| Source of bias | Question | Answer (High, Low, Unclear risk of bias) |
| --- | --- | --- |
| Selection bias | Does the study population deviate from the general population such that the reported outcome (mortality) is at high risk of bias? | Yes= High risk - The study population is a subgroup of the general population e.g. of only HIV patients, patients with malaria, patients with severe typhoid fever, …  No= Low risk - The study population is representative for the general population. |
|  | Are individuals infected with either S. Typhi or S. Paratyphi detected by passive surveillance, introducing possibly bias in the outcome? | Yes= High risk - Passive surveillance relies on reporting of health care providers and may not cover all the cases.  No= Low risk- Active surveillance relies on active case finding by health care providers in the community |
| Attrition bias: Drop-out | Are drop-outs present in the study? | Yes= High risk – The presence of drop-out might introduce bias due to the fact that patients are severely ill and are not able to go to the hospital for follow-up. Another reason for drop-out might be death.  No= Low risk – The absence of drop-out makes sure that the diagnosis of enteric fever and the outcome are reported for all patients |
| Measurement bias | Is the diagnostic test used to diagnose enteric fever likely to introduce bias in the CFR for enteric fever? | Yes= High risk – The methods used to detect enteric fever, resulting in low sensitivity (and specificity) are stool culture and Widal test  No= Low risk – Diagnosis of enteric fever occurs via blood culture, bone marrow culture, Tubex, Typhidot, and PCR |
| Other? | Are there any other important concerns with the paper that were not stated above that could put the reported outcome at high risk of bias? |  |

**Supplement 4: References for Table 2**

A1. Seydi M, Soumare M, Sow AI, Diop BM, Sow PS. Aspects actuels des bactériémies à Salmonella à la clinique des maladies infectieuses Ibrahima Diop Mar du centre hospitalier national de Fann (Sénégal). Med. Mal. Infect. 2005; 35:23–27.

A2. Tohme A, Zein E, Nasnas R. Typhoid fever. Clinical and therapeutic study in 70 patients. Leban. Med. J. 2004; 52:71–77. Available at: http://ovidsp.ovid.com/ovidweb.cgi?T=JS&CSC=Y&NEWS=N&PAGE=fulltext&D=cagh3&AN=20053083765%5Cnhttp://wa4py6yj8t.search.serialssolutions.com/?url_ver=Z39.88-2004&rft_val_fmt=info:ofi/fmt:kev:mtx:journal&rfr_id=info:sid/Ovid:cagh3&rft.genre=article&rft_id=in.

A3. Reynaud R. Les aspects actuels des fièvres typhoparatyphiques en milieu hospitalier et leur évolution sous traitement. Maroc Médical 1971; 548:1445–447.

A4. Papa F, Peyron R. [Salmonella in Algeria: the Salmonella of northern area of the Sahara]. Arch. l’Institut Pasteur d’Algerie. Inst. Pasteur d’Algerie 1970; 48:73–83. Available at: http://www.ncbi.nlm.nih.gov/pubmed/4950342.

A5. Gallais H, Raoult D, De Rego A, Morvan D, Casanova P. La fièvre typhoïde en Afrique Noire. Médecine Trop. Rev. du Corps santé Colon. 983; 43:367–70.

A6. Derrien J-P, N’Doye B, Gaultier Y, Le Bris H, Varieras G, Thomas J. Les salmonelloses à l’Hôpital Principal de Dakar en 1977. Dakar Médical 1979; 24:6–11.

A7. Lefebvre N, Gning SB, Nabeth P, et al. [Clinical and laboratory features of typhoid fever in Senegal. A 70-case study]. Médecine Trop. Rev. du Corps santé Colon. 2005; 65:543–548. Available at: http://ovidsp.ovid.com/ovidweb.cgi?T=JS&PAGE=reference&D=med4&NEWS=N&AN=16555513.

A8. Mendoza-Hernandez P, Terminel-Valenzuela M, Ruiz-Maya L. Experiencias bacteriologicas, clinicas y terapeuticas en 1676 casos de fiebre tifoidea. Gac. Med. Mex. 1974; 108:89–92.

A9. Butler T, Rumans L, Arnold K. Response of Typhoid Fever Caused by Chloramphenicol-Susceptible and Chloramphenicol- Resistant Strains of Salmonella typhi to Treatment with Trimethoprim-Sulfamethoxazole Author ( s ): Thomas Butler , Larry Rumans and Keith Arnold Source : Reviews of Infe. Rev. Infect. Dis. 1982; 4:551–561.

A10. Van Den Bergh ETAM, Hussein Gasem M, Keuter M, Dolmans M V. Outcome in three groups of patients with typhoid fever in Indonesia between 1948 and 1990. Trop. Med. Int. Heal. 1999; 4:211–215.

A11. Rao PS, Rajashekar V, Varghese GK, Shivananda PG. Emergence of multidrug-resistant {Salmonella} typhi in rural southern {India}. Am. J. Trop. Med. Hyg. 1993; 48:108–111.

A12. Maskey AP, Day JN, Tuan PQ, et al. Salmonella enterica Serovar Paratyphi A and S. enterica Serovar Typhi Cause Indistinguishable Clinical Syndromes in Kathmandu, Nepal. Clin. Infect. Dis. 2006; 42:1247–1253. Available at: https://academic.oup.com/cid/article-lookup/doi/10.1086/503033.

A13. Erma Abucejo P, Rosario Capeding M, Lupisan SP, et al. Blood culture confirmed typhoid fever in a provincial hospital in the Philippines. Southeast Asian J. Trop. Med. Public Health 2001; 32:531–536.

A14. Brown GW, Shirai A, Jegathesan M, et al. Febrile illness in Malaysia - An analysis of 1629 hospitalized patients. Am. J. Trop. Med. Hyg. 1984; 33:311–315.

A15. Hoa NTT, Diep TS, Wain J, et al. Community-acquired septicaemia in southern Viet Nam: the importance of multidrug-resistant Salmonella typhi. Trans. R. Soc. Trop. Med. Hyg. 1998; 92:503–508.

A16. Kabir S, Azhar MA, Ekram ARMS, Islam QT, Ahmed I. Current Clinical Profile of Enteric Fever in a Teaching Hospital. 2002; 15:5–7.

A17. Khosla SN, Saman A, Khosla P, et al. Drug resistant typhoid fever. Trop. Doct. 1998; 28:235–237.

A18. Koh TS, Goh KT. Enteric fever surveillance in Singapore. Singapore Med. J. 1976; 17:32–37.

A19. Lin FYC, Ho VA, Bay P V., et al. The epidemiology of typhoid fever in the Dong Thap Province, Mekong Delta region of Vietnam. Am. J. Trop. Med. Hyg. 2000; 62:644–648.

A20. Mathur GM, Sharma R. A study of ttyphoid fever in Jaipur, India. Trop. Geogr. Med. 1971; 23:329–334.

A21. Mukherjee P, Mukherjee S, Dalal BK, Haldar KK, Ghosh E, Pal TK. Some prospective observations on recent outbreak of typhoid fever in West Bengal. J. Assoc. Physicians India 1991; 39:445–448.

A22. Parande MA, Patil CG, Rayate M V, Lukde MU. Study of Clinical Profile, Complications and Response to Treatment of Enteric Fever Cases Admitted in a Tertiary Care Hospital of Solapur. Indian J. Public Heal. Res. Dev. 2015; 6:61. Available at: http://www.indianjournals.com/ijor.aspx?target=ijor:ijphrd&volume=6&issue=1&article=011.

A23. Parry CM, Thompson C, Vinh H, et al. Risk factors for the development of severe typhoid fever in Vietnam. BMC Infect. Dis. 2014; 14:73. Available at: http://bmcinfectdis.biomedcentral.com/articles/10.1186/1471-2334-14-73.

A24. Phetsouvanh R, Phongmany S, Soukaloun D, et al. Causes of community-acquired bacteremia and patterns of antimicrobial resistance in Vientiane, Laos. Am. J. Trop. Med. Hyg. 2006; 75:978–985.

A25. Sen SK, Mahakur AC. Enteric fever-A comparative study of adult and paediatric cases. Indian J. Pediatr. 1972; 39:354–360.

A26. Shahunja KM, Leung DT, Ahmed T, et al. Factors Associated with Non-typhoidal Salmonella Bacteremia versus Typhoidal Salmonella Bacteremia in Patients Presenting for Care in an Urban Diarrheal Disease Hospital in Bangladesh. PLoS Negl. Trop. Dis. 2015; 9:1–12.

A27. Walia M, Gaind R, Mehta R, Paul P, Aggarwal P, Kalaivani M. Current perspectives of enteric fever: a hospital-based study from India. Ann. Trop. Paediatr. 2005; 25:161–74. Available at: http://www.ncbi.nlm.nih.gov/pubmed/16156980.

A28. Abdurrahman MB, Joss D V. Pattern of enteric fever in Kaduna, Nigeria. Niger. Med. J. 1979; 9:437–441. Available at: http://ovidsp.ovid.com/ovidweb.cgi?T=JS&CSC=Y&NEWS=N&PAGE=fulltext&D=cagh0&AN=19812902000%5Cnhttp://wa4py6yj8t.search.serialssolutions.com/?url_ver=Z39.88-2004&rft_val_fmt=info:ofi/fmt:kev:mtx:journal&rfr_id=info:sid/Ovid:cagh0&rft.genre=article&rft_id=in.

A29. Abraham G, Teklu B. Typhoid fever: clinical analysis of 50 Ethiopian patients. Ethiop. Med. J. 1981; 19:41–46. Available at: http://ovidsp.ovid.com/ovidweb.cgi?T=JS&CSC=Y&NEWS=N&PAGE=fulltext&D=cagh0&AN=19822901721%5Cnhttp://wa4py6yj8t.search.serialssolutions.com/?url_ver=Z39.88-2004&rft_val_fmt=info:ofi/fmt:kev:mtx:journal&rfr_id=info:sid/Ovid:cagh0&rft.genre=article&rft_id=in.

A30. Akinyemi KO, Oshundare YO, Oyeyinka OG, Coker AO. A retrospective study of community-acquired Salmonella infections in patients attending public hospitals in Lagos, Nigeria. J. Infect. Dev. Ctries. 2012; 6:387–395. Available at: http://www.jidc.org/index.php/journal/article/view/2120.

A31. Ameh IG, Opara WEK. Typhoid: a record of cases in Sokoto, Nigeria. Pakistan J. Biol. Sci. 2004; 7:1177–1180. Available at: http://ovidsp.ovid.com/ovidweb.cgi?T=JS&CSC=Y&NEWS=N&PAGE=fulltext&D=cagh3&AN=20043120657%5Cnhttp://oxfordsfx.hosted.exlibrisgroup.com/oxford?sid=OVID:caghdb&id=pmid:&id=doi:10.3923%2Fpjbs.2004.1177.1180&issn=1028-8880&isbn=&volume=7&issue=7&spage=1177&pa.

A32. Breiman RF, Cosmas L, Njuguna H, et al. Population-based incidence of typhoid fever in an urban informal settlement and a rural area in Kenya: Implications for typhoid vaccine use in Africa. PLoS One 2012; 7.

A33. Elegbeleye OO. Typhoid fever in Lagos - Nigeria. West Indian Med. J. 1976; 15:39–42.

A34. Feasey NA, Gaskell K, Wong V, et al. Rapid Emergence of Multidrug Resistant, H58-Lineage Salmonella Typhi in Blantyre, Malawi. PLoS Negl. Trop. Dis. 2015; 9:1–13.

A35. Keddy KH, Sooka A, Smith AM, et al. Typhoid fever in South Africa in an endemic HIV setting. PLoS One 2016; 11:1–12.

A36. Popkiss ME. Typhoid fever: A report on a point-source outbreak of 69 cases in Cape Town. 1980; 57:325–329. Available at: http://ovidsp.ovid.com/ovidweb.cgi?T=JS&PAGE=reference&D=med2&NEWS=N&AN=7355351.

A37. Weeramanthri TS, Corrah PT, Mabey DCW, Greenwood BM. Clinical experience with enteric fever in The Gambia, West Africa 1981-1986. J. Trop. Med. Hyg. 1989; 92:272–275. Available at: http://www.embase.com/search/results?subaction=viewrecord&from=export&id=L19205537.

A38. Wicks ACB, Holmes GS, Davidson L. Endemic typhoid fever. Q. J. Med. 1971; 40:341–354. Available at: http://ovidsp.ovid.com/ovidweb.cgi?T=JS&CSC=Y&NEWS=N&PAGE=fulltext&D=cagh0&AN=19722701152%5Cnhttp://wa4py6yj8t.search.serialssolutions.com/?url_ver=Z39.88-2004&rft_val_fmt=info:ofi/fmt:kev:mtx:journal&rfr_id=info:sid/Ovid:cagh0&rft.genre=article&rft_id=in.

A39. Grell GAC, M.B., B.S., M.R.C.P., D.T.M., H. Typhoid fever in Dominica, W.I. - Report on the clinical features of 78 cases. West Indian Med. J. 1979; 28:94.

A40. Macfarlane DE, Narla VR. Bacteraemia at the University Hospital of the West Indies - a report of 222 cases. J. Infect. 1985; 10:126–142.

**Supplement 5: Leave-one-out sensitivity analysis**

**Supplementary Table 2: Leave-one-out sensitivity analysis.**

| Study left out^a,b^ | CFR | 95% CI Lower limit | 95% CI Upper limit | 95% PI Lower limit | 95% PI Upper limit | Tau | I² |
| --- | --- | --- | --- | --- | --- | --- | --- |
| Abdurrahman [A28] | 0.0239 | 0.0157 | 0.036 | 0.0024 | 0.1969 | 1.1575 | 0.9456 |
| Abraham [A29] | 0.0239 | 0.0158 | 0.0362 | 0.0024 | 0.1999 | 1.1654 | 0.9474 |
| Abucejo [A13] | 0.0249 | 0.0162 | 0.038 | 0.0024 | 0.2164 | 1.1958 | 0.9496 |
| Akinyemi [A30] | 0.0259 | 0.0171 | 0.0392 | 0.0026 | 0.2117 | 1.1602 | 0.9231 |
| Ameh [A31] | 0.0252 | 0.0165 | 0.0383 | 0.0024 | 0.2161 | 1.1886 | 0.9491 |
| Breiman [A32] | 0.0262 | 0.0175 | 0.0391 | 0.0028 | 0.2057 | 1.1364 | 0.9454 |
| Brown [A14] | 0.0251 | 0.0165 | 0.0382 | 0.0024 | 0.2148 | 1.186 | 0.9495 |
| Butler (Jakarta) [A9] | 0.0243 | 0.016 | 0.037 | 0.0023 | 0.2092 | 1.1856 | 0.9495 |
| Butler (Saigon) [A9] | 0.0245 | 0.0161 | 0.0371 | 0.0024 | 0.2087 | 1.1815 | 0.9492 |
| Derrien [A6] | 0.0244 | 0.016 | 0.0371 | 0.0023 | 0.2113 | 1.191 | 0.9498 |
| Elegbeleye [A33] | 0.0237 | 0.0159 | 0.0353 | 0.0027 | 0.1803 | 1.1056 | 0.9415 |
| Feasey [A34] | 0.0248 | 0.0162 | 0.0378 | 0.0023 | 0.2162 | 1.1977 | 0.9496 |
| Gallais [A5] | 0.0245 | 0.016 | 0.0374 | 0.0023 | 0.2141 | 1.1973 | 0.9498 |
| Grell [A39] | 0.0253 | 0.0166 | 0.0382 | 0.0025 | 0.2136 | 1.1794 | 0.9491 |
| Hoa [A39] | 0.0263 | 0.0175 | 0.0393 | 0.0028 | 0.2062 | 1.1369 | 0.9454 |
| Kabir [A16] | 0.0248 | 0.0162 | 0.0376 | 0.0024 | 0.2141 | 1.1919 | 0.95 |
| Keddy [A35] | 0.0241 | 0.0158 | 0.0367 | 0.0023 | 0.2077 | 1.1859 | 0.9481 |
| Khosla [A17] | 0.0241 | 0.0158 | 0.0367 | 0.0023 | 0.2081 | 1.1866 | 0.9486 |
| Koh [A18] | 0.0249 | 0.0163 | 0.038 | 0.0024 | 0.2168 | 1.1962 | 0.9485 |
| Lefebvre [A7] | 0.0252 | 0.0166 | 0.0382 | 0.0025 | 0.2137 | 1.1811 | 0.9492 |
| Lin [A19] | 0.0257 | 0.0171 | 0.0386 | 0.0026 | 0.2098 | 1.1588 | 0.9474 |
| Macfarlane [A40] | 0.0252 | 0.0167 | 0.038 | 0.0025 | 0.2111 | 1.1728 | 0.9486 |
| Maskey (*S*. Typhi) [A12] | 0.0271 | 0.0184 | 0.0398 | 0.0032 | 0.1952 | 1.086 | 0.9405 |
| Maskey (*S*. Paratyphi) [A12] | 0.0265 | 0.0178 | 0.0393 | 0.0029 | 0.2028 | 1.1218 | 0.9441 |
| Mathur [A20] | 0.0239 | 0.0157 | 0.036 | 0.0024 | 0.1977 | 1.16 | 0.9234 |
| Mendoza-Hernandez [A8] | 0.0245 | 0.0159 | 0.0373 | 0.0023 | 0.2146 | 1.1993 | 0.9446 |
| Mukherjee [A21] | 0.0239 | 0.0158 | 0.0361 | 0.0024 | 0.1984 | 1.1611 | 0.9471 |
| Papa [A4] | 0.0246 | 0.016 | 0.0375 | 0.0023 | 0.2151 | 1.1986 | 0.9496 |
| Parande [A22] | 0.0251 | 0.0164 | 0.0381 | 0.0024 | 0.2154 | 1.1887 | 0.9496 |
| Parry [A23] | 0.0262 | 0.0174 | 0.0393 | 0.0027 | 0.208 | 1.1443 | 0.9458 |
| Phetsouvanh [A24] | 0.0253 | 0.0166 | 0.0384 | 0.0024 | 0.215 | 1.1836 | 0.9492 |
| Popkiss [A36] | 0.0258 | 0.0171 | 0.0386 | 0.0026 | 0.2095 | 1.1571 | 0.9472 |
| Rao (*S*. Typhi) [A11] | 0.0254 | 0.0168 | 0.0384 | 0.0025 | 0.213 | 1.1743 | 0.9486 |
| Rao (*S*. Paratyphi) [A11] | 0.0254 | 0.0168 | 0.0383 | 0.0025 | 0.211 | 1.1686 | 0.9482 |
| Reynaud [A3] | 0.0245 | 0.016 | 0.0374 | 0.0023 | 0.2148 | 1.1988 | 0.9492 |
| Sen [A25] | 0.0251 | 0.0165 | 0.038 | 0.0024 | 0.2138 | 1.1842 | 0.9495 |
| Seydi [A1] | 0.0237 | 0.0159 | 0.0354 | 0.0026 | 0.1842 | 1.1184 | 0.9431 |
| Shahunja [A26] | 0.0258 | 0.0171 | 0.0386 | 0.0026 | 0.2096 | 1.1575 | 0.9473 |
| Tohme [A2] | 0.0252 | 0.0166 | 0.0382 | 0.0025 | 0.2137 | 1.1811 | 0.9492 |
| Van Den Bergh (Yogyakarta) [A10] | 0.024 | 0.0158 | 0.0364 | 0.0024 | 0.2033 | 1.1744 | 0.9482 |
| Van Den Bergh (Semarang) [A10] | 0.0244 | 0.0159 | 0.0372 | 0.0023 | 0.2123 | 1.194 | 0.9499 |
| Walia [A27] | 0.0244 | 0.016 | 0.0372 | 0.0023 | 0.2127 | 1.194 | 0.95 |
| Weeramanthri [A37] | 0.0245 | 0.016 | 0.0374 | 0.0023 | 0.2134 | 1.1938 | 0.95 |
| Wicks [A38] | 0.0241 | 0.0158 | 0.0366 | 0.0023 | 0.2071 | 1.1846 | 0.9479 |

^a^Round brackets after the author indicates evaluation for the specific study or Salmonella serovar when multiple studies or Salmonella serovars are described in one article; ^b^The full citations of the articles are provided in Supplement 2.

**Supplement 6: Subgroup analyses**

In order to investigate the heterogeneity in the overall CFR, we conducted subgroup analyses. We categorized studies according to WHO-region, World Bank income category, detection method used, *Salmonella* serovar included in the study, and presence of HIV-infected individuals in the study population. As an exploratory analysis, we performed a meta-regression including the variable under investigation to assess potential heterogeneity in CFR according to their categories.

The full citations of the included articles are provided in Supplement 2.


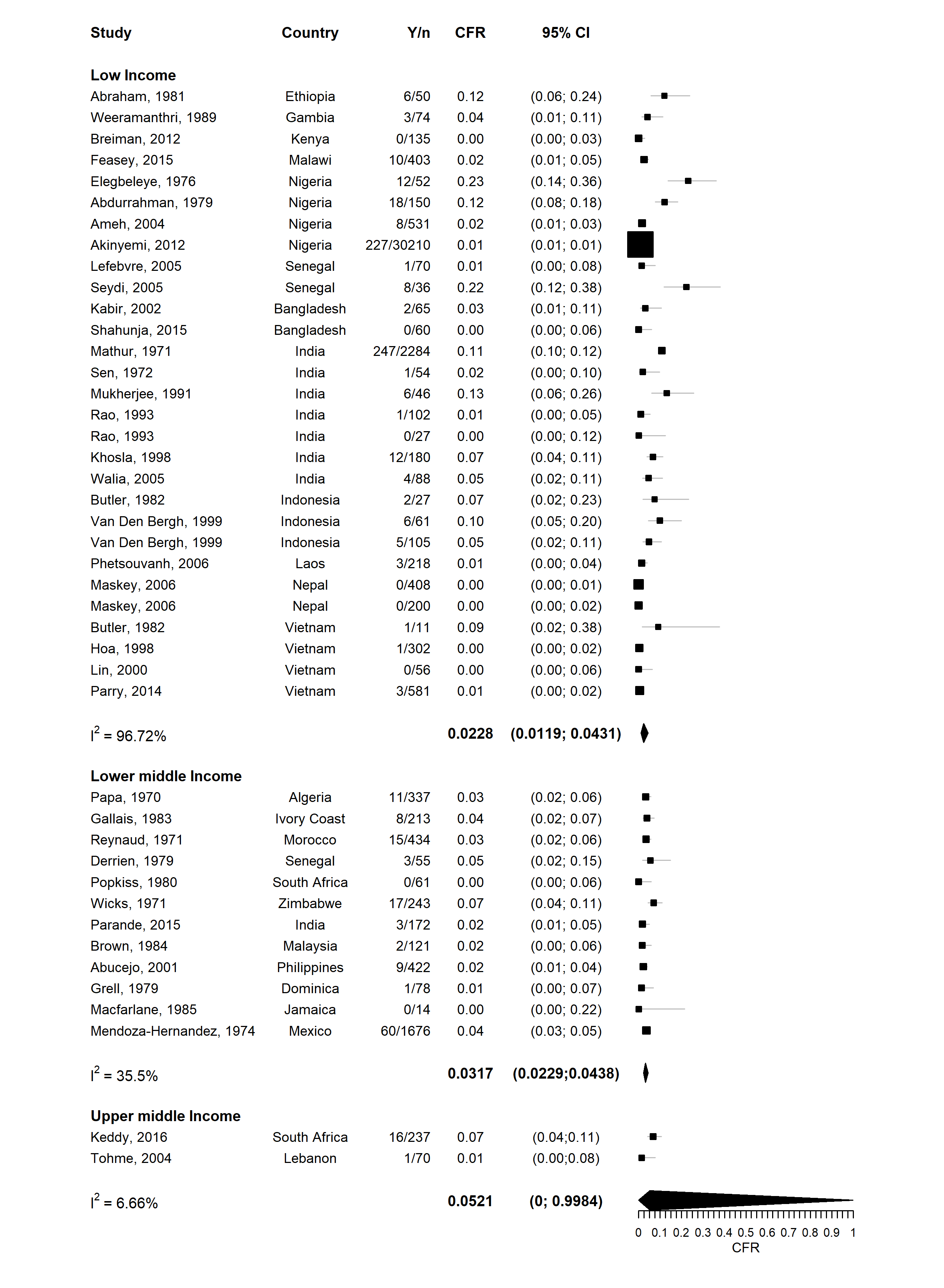


**Supplementary Figure 4: Forest plot for the CFR of enteric fever according to World Bank Income Category.**

Only one study (Koh and colleagues) was classified as a having a high income and was not plotted in the figure [A18]. The overall estimate for each subgroup was obtained from a random intercept logistic regression model. The 95% CI of the individual studies were Wilson Score intervals, while the CI of the overall estimate for each subgroup was based on a t-distribution. n=number of cases, Y= number of deaths.

S

We tested whether there was a difference in CFR between the WHO regions, which included the study of Koh and colleagues [A18]. However, we found that the income level did not influence the estimated average CFR.


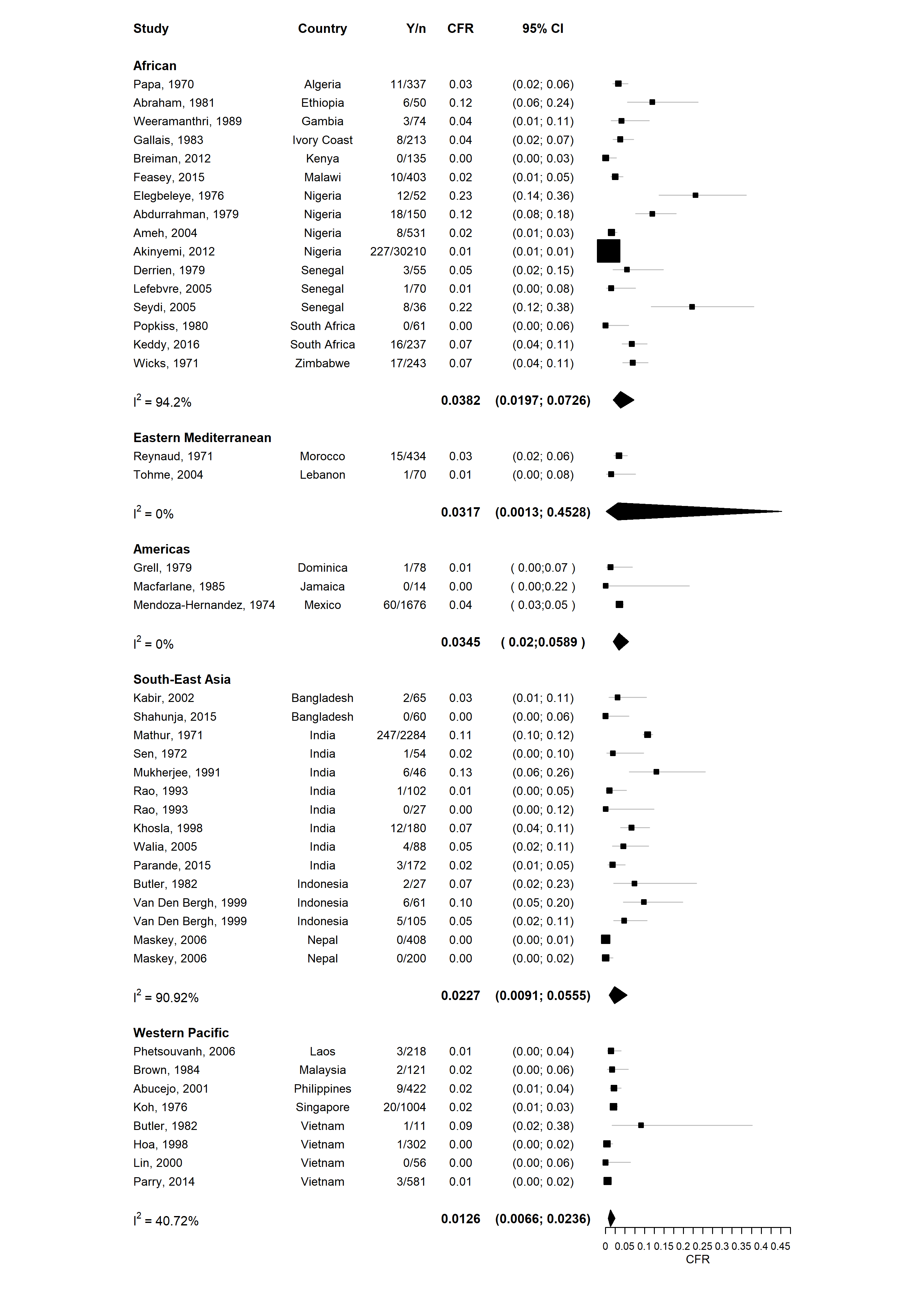


**Supplementary Figure 5: Forest plot for the CFR of enteric fever according to WHO region.**

The overall estimate for each subgroup was obtained from a random intercept logistic regression model. The 95% CI of the individual studies were Wilson Score intervals, while the CI of the overall estimate for each subgroup was based on a t-distribution. n=number of cases, Y= number of deaths.

We conducted a meta-regression which included the variable WHO region. We found that WHO region does not significantly influence the estimated average CFR. However, the Western Pacific region showed a significant effect on the CFR, suggesting the CFR is significantly different (lower) in this region.


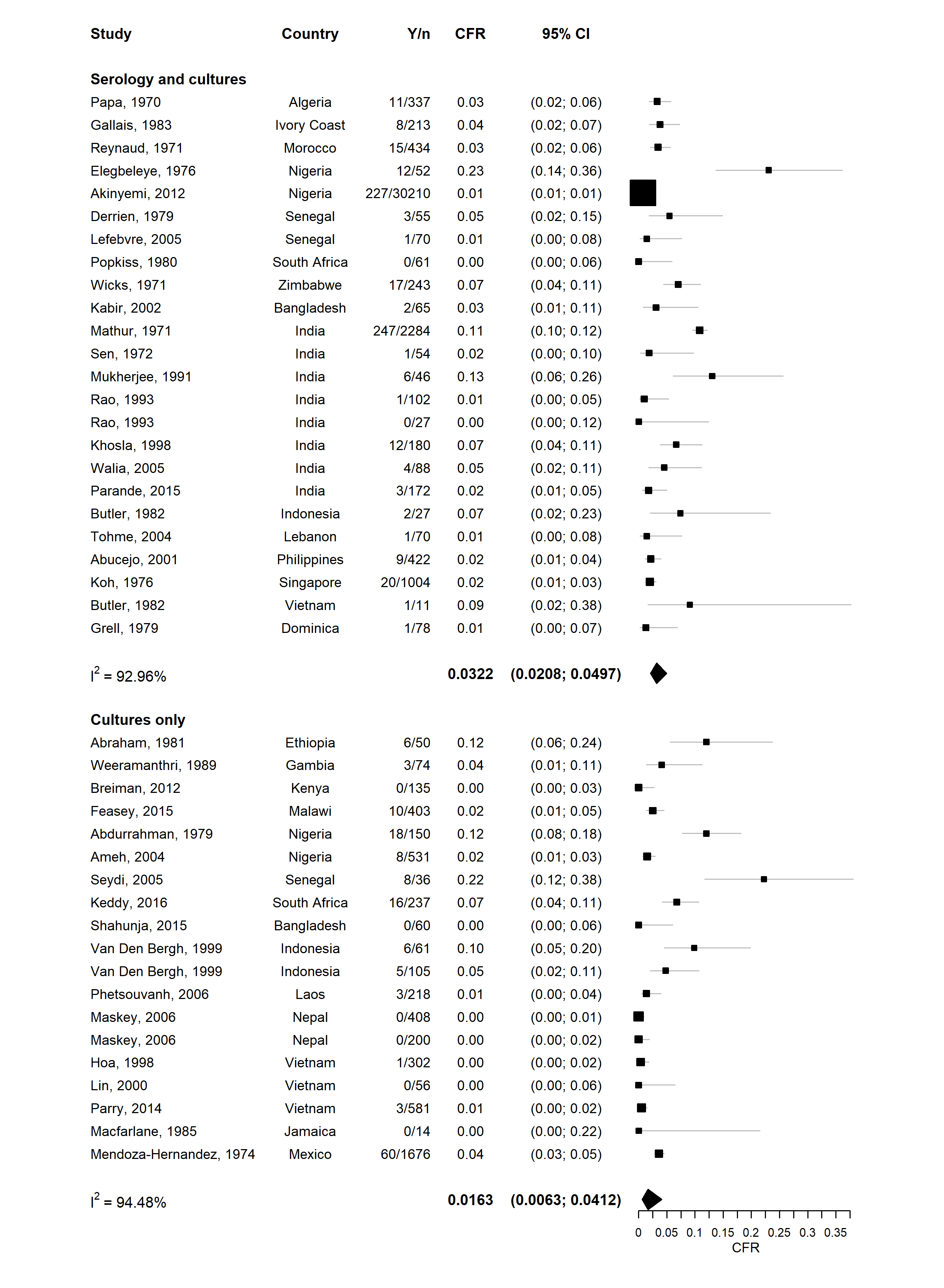


**Supplementary Figure 6: Forest plot for the CFR of enteric fever according to detection method.**

The study from Brown and colleagues was not included since they only used a serological test to identify cases [A14]. The overall estimate for each subgroup was obtained from a random intercept logistic regression model. The 95% CI of the individual studies were Wilson Score intervals, while the CI of the overall estimate for each subgroup was based on a t-distribution. n=number of cases, Y= number of deaths.

When the detection method was added to the model, we found that it did not significantly influence the estimated average CFR.


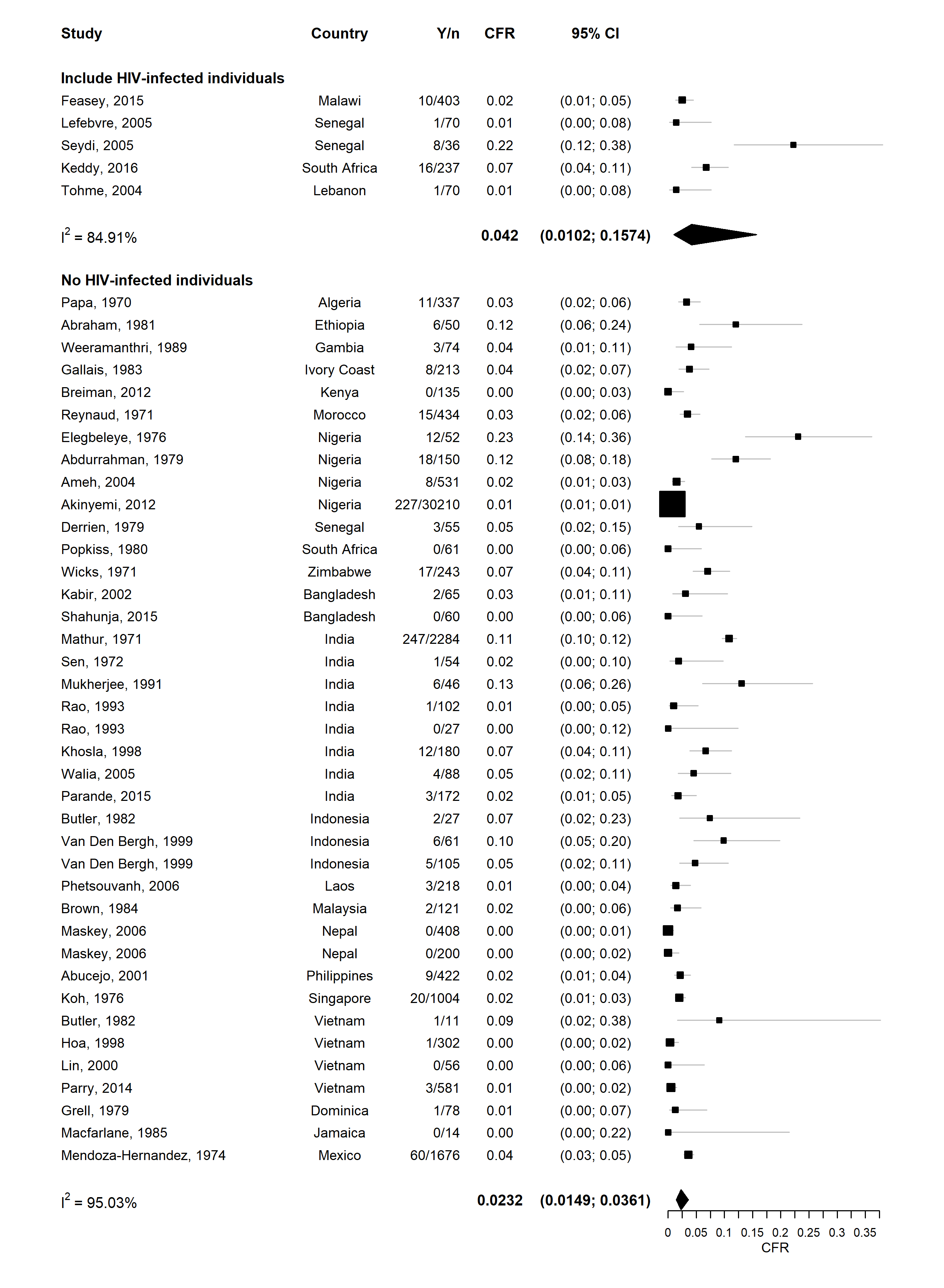


**Supplementary Figure 7: Forest plot for the CFR of enteric fever according to the inclusion of HIV-infected individuals in the study population.**

The overall estimate for each subgroup was obtained from a random intercept logistic regression model. The 95% CI of the individual studies were Wilson Score intervals, while the CI of the overall estimate for each subgroup was based on a t-distribution. n=number of cases, Y= number of deaths.

We found that HIV status did not significantly influence the estimated average CFR.

**Supplementary Figure 8: Forest plot for the CFR of enteric fever according to the *Salmonella* serovar(s) included in the study.**


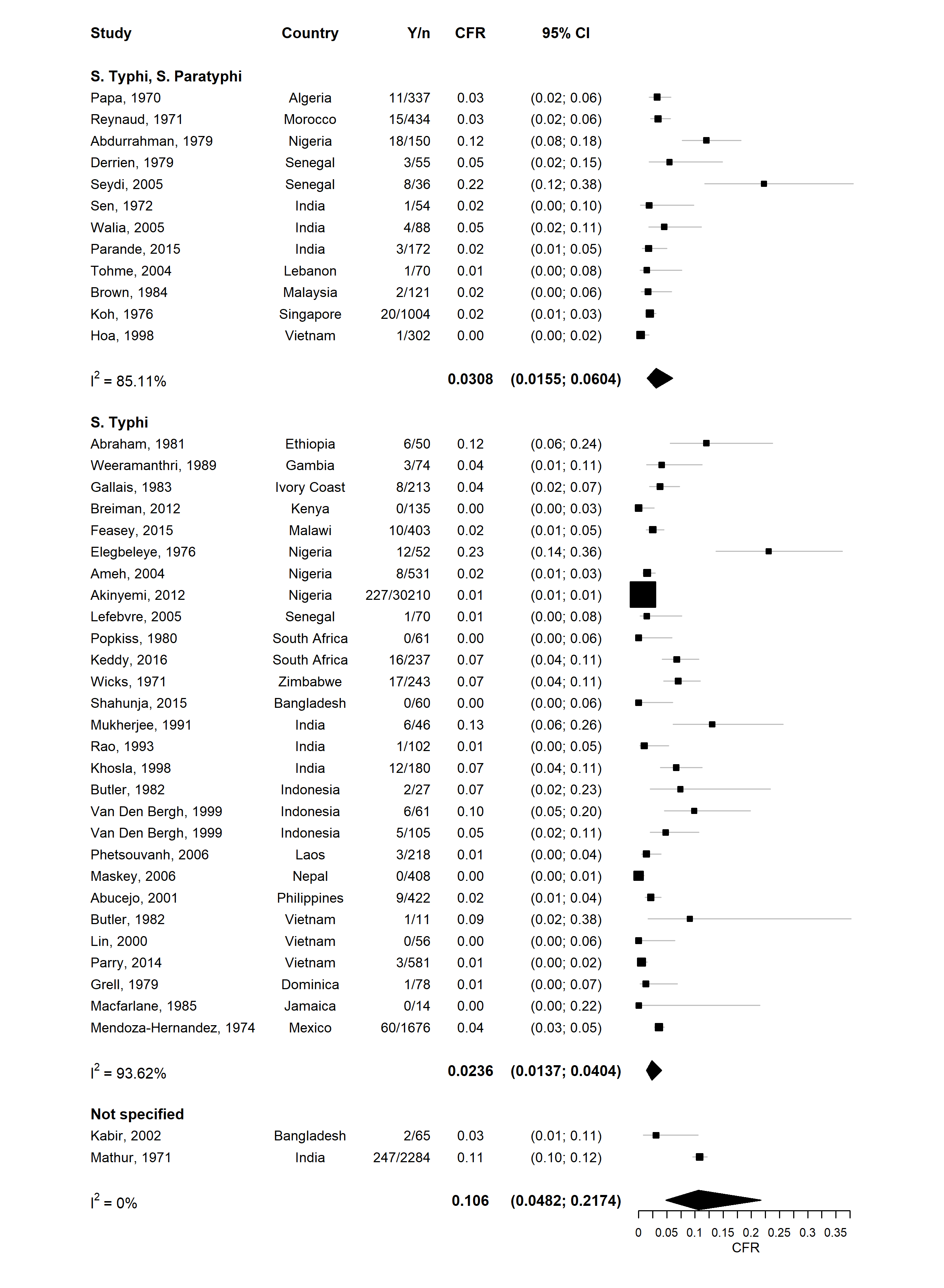


The two studies reporting the CFR for *S*. Paratyphi were not included due to model fitting problems [A11,A12]. The overall estimate for each subgroup was obtained from a random intercept logistic regression model. The 95% CI of the individual studies were Wilson Score intervals, while the CI of the overall estimate for each subgroup was based on a t-distribution. n=number of cases, Y= number of deaths.

We were not able to perform a meta-regression including the covariate *Salmonella* serovar due to model fitting issues.


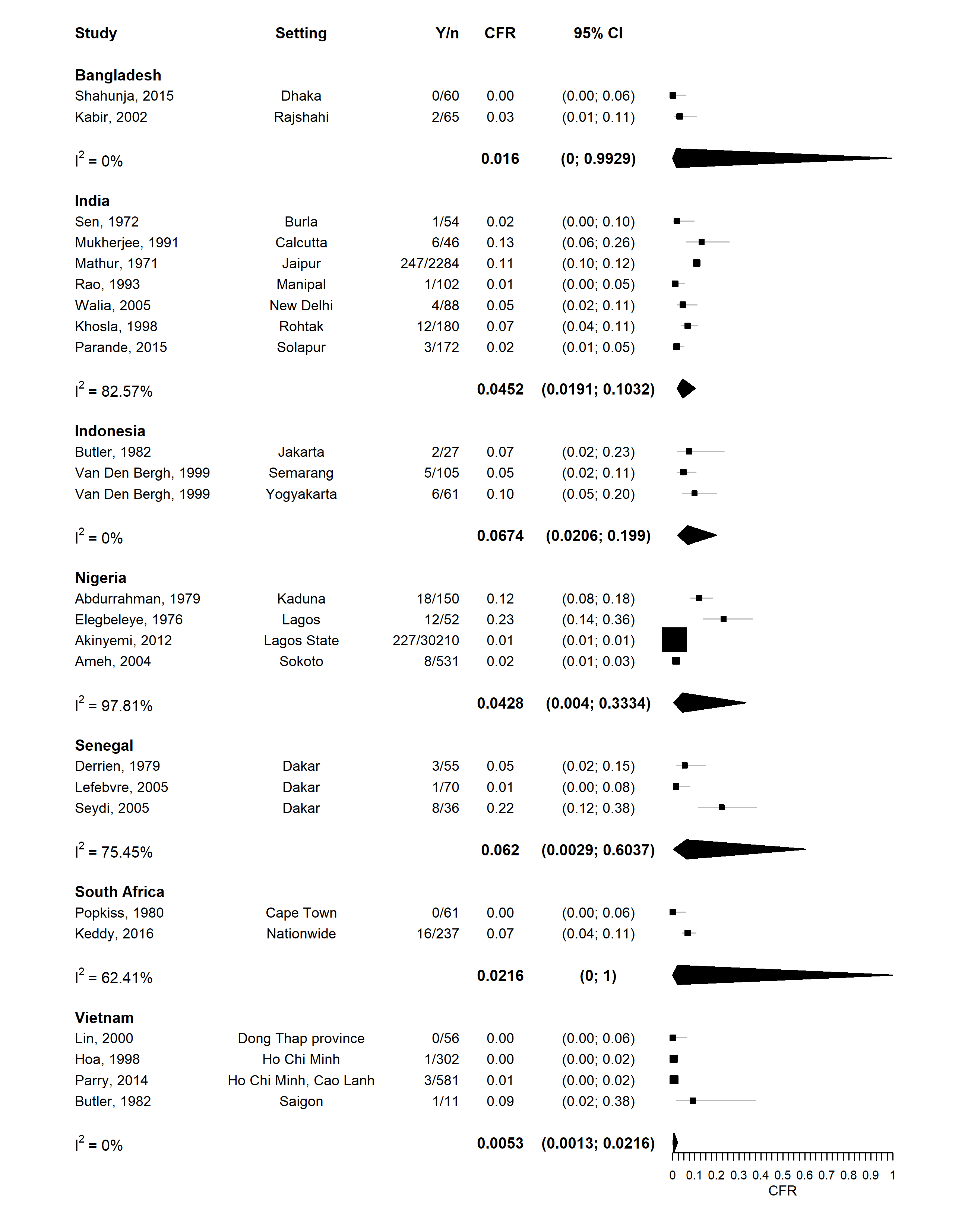


**Supplementary Figure 9: Forest plot for the CFR of enteric fever according to countries with multiple estimates.**

The overall estimate for each subgroup was obtained from a random intercept logistic regression model. The 95% CI of the individual studies were Wilson Score intervals, while the CI of the overall estimate for each subgroup was based on a t-distribution. n=number of cases, Y= number of deaths.

**Supplement 7: Investigation of prognostic factors**

**Supplementary Table 3: Overview of studies including mortality for different age groups**

|  |  | ≤15 years^a^ | | >15 years^a^ | |  |
| --- | --- | --- | --- | --- | --- | --- |
| Author^b^ | **Year** | $Y_{i}$ | $n_{i}$ | $Y_{i}$ | $n_{i}$ | **Remarks** |
| Abdurrahman [A28] | 1979 | 8 | 89 | 10 | 60 | 1 patient with unknown age which is not included in these estimates |
| Abucejo [A13] | 2001 | 2 | 153 | 7 | 268 | Number of individuals belonging to each age group were extracted from Figure 2 using WebPlotDigitizer.[8] |
| Akinyemi [A30] | 2012 | 102 | 6850 | 125 | 23360 | Table 5: age group 50 and above contains 7 individuals (Contacted Author) |
| Feasey [A34] | 2015 | 7 | 330 | 3 | 73 | Author mentions children and adult, we assumed a cutoff of 16 years old. |
| Grell [A39] | 1979 | 0 | 53 | 1 | 25 |  |
| Hoa [A15] | 1998 | 0 | 133 | 1 | 169 | Outcome is known for 302/309 patients, we assumed that in both groups the outcome is known for 97·7% of the cases. |
| Mathur [A20] | 1971 | 106 | 825 | 141 | 1459 |  |
| Parry [A23] | 2014 | 0 | 355 | 3 | 226 | Number of individuals belonging to each age group were extracted from Figure 1 using WebPlotDigitizer.[8] |
| Popkiss [A36] | 1980 | 0 | 21 | 0 | 40 |  |
| Walia [A27] | 2005 | 3 | 68 | 1 | 20 |  |
| Lefebvre [A7] | 2005 | 0 | 37 | 1 | 33 | Figure 1 reporting number of individuals in each age category is interpreted as follows: [1-5[: 7, [5-10[: 15, [10-15[: 15, [15-20[: 14, [20-25[: 9, [25-30[: 1, [30-35[: 5, [35-40[: 0, [40-45[: 1, [45-50[: 3 |
| Mendoza-Hernandez [A8] | 1974 | 15 | 574 | 45 | 1102 |  |

^a^Age bins were pre-specified and studies reporting mortality according to age groups were classified in either bins for which the age groups have the highest coverage; ^b^The full citations of the articles are provided in Supplement 2.

**Supplementary Table 4: Overview of studies reporting mortality for antibiotic resistance**

| Author^a,b^ | Year | $\boldsymbol{Y}_{\boldsymbol{i}}$ | $\boldsymbol{n}_{\boldsymbol{i}}$ | Antibiotics tested | Antibiotics resistance observed | Remarks |
| --- | --- | --- | --- | --- | --- | --- |
| Abucejo [A13] | 1979 | 0 | 0 | 1^st^ and 2^nd^ line antibiotics^c^ | - |  |
| Butler (Jakarta) [A9] | 1982 | 0 | 0 | Amp, Ceph, Chlor, Col, Gent, Tetra, TMP-SMZ | - | Only 39/60 isolates could be tested for antimicrobial sensitivity |
| Butler (Saigon) [A9] | 1982 | 0 | 7 | Amp, Ceph, Col, Chlor, Tetra, Strep, Sulf | Chlor, Strep, Sulfo, Tetra | Only 13/20 isolates could be tested for antimicrobial sensitivity |
| Keddy [A35] | 2016 | 5 | 55 | Amp, TMP-SMZ, Chlor, Tetra, Cipro, Ceft | MDR was defined as resistance to 3 or more of the tested antimicrobials | Mortality could only be recorded for 55/158 patients with MDR S. Typhi |
| Khosla [A17] | 1998 | 10 | 124 | 1^st^ line antibiotics, Tetra, Amo, Gent, Cipro | Chlor, Chlor+Cotrim+Tetra, Chlor+Cotrim+Amp, Chlor+Cotrim+Cipro,  Tetra+Cotrim+Amp+Gent |  |
| Mukherjee [A21] | 1991 | 5 | 31 | - | Chlor |  |
| Rao  (*S*. Typhi) [A11] | 1993 | 1 | 80 | Chlor, Amp, Tetra, Gent, Kan, Amo, TMP-SMZ, Meth, Cipro, Nor | Chlor, Amp, TMP-SMZ |  |
| Rao  (*S*. Paratyphi) [A11] | 1993 | 0 | 0 | Chlor, Amp, Tetra, Gent, Kan, Amo, TMP-SMZ, Meth, Cipro, Nor | - |  |
| Walia [A27] | 2005 | 2 | 26 | Ceft, TMP-SMZ, Cefi, Cipro, Nal  1^st^ line antibiotics, Nal | MDR was defined as resistant to Amp, Chlor and Cotrim | NAR was defined as resistant to Nal, but these numbers were not used here |
| Weeramanthri [A37] | 1989 | 0 | 0 | Chlor, Amp, Tetra, Sep | - |  |
| Derrien [A6] | 1979 | 0 | 0 | Chlor | - |  |
| Papa [A4] | 1970 | 0 | 0 | Not specified | - |  |

^a^Round brackets after the author indicates evaluation for the specific study or Salmonella serovar when multiple studies or Salmonella serovars are described in one article; ^b^The full citations of the articles are provided in Supplement 2; ^c^1st line antibiotics: Chloramphenicol (Chlor), Co-trimoxazole (Cotrim), Ampicillin (Amp); 2^nd^ line antibiotics: Ceftriaxon (Ceft), Ciprofloxacin (Cipro), Ofloxacin (Ofl); Cephalothin (Ceph), Colistin (Col), Gentamicin (Gent), Tetracycline (Tetra) , Trimethoprim-Sulfamethoxazole (TMP-SMZ), Streptomycin (Strep), Sulfonamides (Sulf), Amoxycilin (Amo), Kanamycin (Kan), Methoxazole (Meth), Norfloxacin (Nor), Nalidixic acid (Nal), Cefixime (Cefi), Septrin (Sep).

**Supplement 8: Individual risk of bias assessment**

**Supplementary Table 5: Individual risk of bias assessment**

| **Author^a,b^** | **Study population** | **Surveillance** | **Attrition** | **Measurement** |
| --- | --- | --- | --- | --- |
| **Abdurrahman** [A28] | ? | + | + | + |
| **Abraham** [A29] | ? | ? | - | - |
| **Abucejo** [A13] | - | + | - | - |
| **Akinyemi** [A30] | - | + | - | + |
| **Ameh** [A31] | - | + | - | + |
| **Breiman** [A32] | - | - | - | - |
| **Brown** [A14] | - | + | + | + |
| **Butler (Jakarta)** [A9] | ? | + | - | + |
| **Butler (Saigon)** [A9] | ? | ? | - | + |
| **Derrien** [A6] | + | + | - | + |
| **Elegbeleye** [A33] | ? | + | - | + |
| **Feasey** [A34] | - | + | + | - |
| **Gallais** [A5] | + | + | - | + |
| **Grell** [A39] | + | + | - | + |
| **Hoa** [A15] | - | + | + | - |
| **Kabir** [A16] | ? | + | + | + |
| **Keddy** [A35] | - | - | + | + |
| **Khosla** [A17] | ? | + | - | + |
| **Koh** [A18] | - | + | - | + |
| **Lefebvre** [A7] | + | + | - | + |
| **Lin** [A19] | - | + | - | - |
| **Macfarlane** [A40] | ? | + | - | - |
| **Maskey** [A12] | - | + | - | - |
| **Mathur** [A20] | - | + | - | + |
| **Mendoza-Hernandez** [A8] | - | + | - | + |
| **Mukherjee** [A21] | - | + | - | - |
| **Papa** [A4] | ? | + | - | + |
| **Parande** [A22] | ? | + | + | + |
| **Parry** [23] | - | + | - | - |
| **Phetsouvanh** [A24] | ? | + | + | - |
| **Popkiss** [A36] | + | - | - | + |
| **Rao** [A11] | ? | + | - | - |
| **Reynaud** [A3] | ? | + | - | + |
| **Sen** [A25] | ? | ? | - | + |
| **Seydi** [A1] | ? | + | - | - |
| **Shahunja** [A26] | + | + | - | + |
| **Tohme** [A2] | - | + | - | + |
| **Van Den Bergh (Yogyakarta)** [A10] | ? | + | - | - |
| **Van Den Bergh (Semarang)** [A10] | ? | + | - | - |
| **Walia** [A27] | - | + | + | - |
| **Weeramanthri** [A37] | ? | + | + | - |
| **Wicks** [A38] | ? | + | + | + |

^a^Round brackets after the author indicates evaluation for the specific study when multiple studies are described in one article; ^b^The full citations of the articles are provided in Supplement 2.

Red indicates high risk of bias, green low risk of bias, and yellow indicates unclear risk of bias.

**Supplement 9: Publication bias**


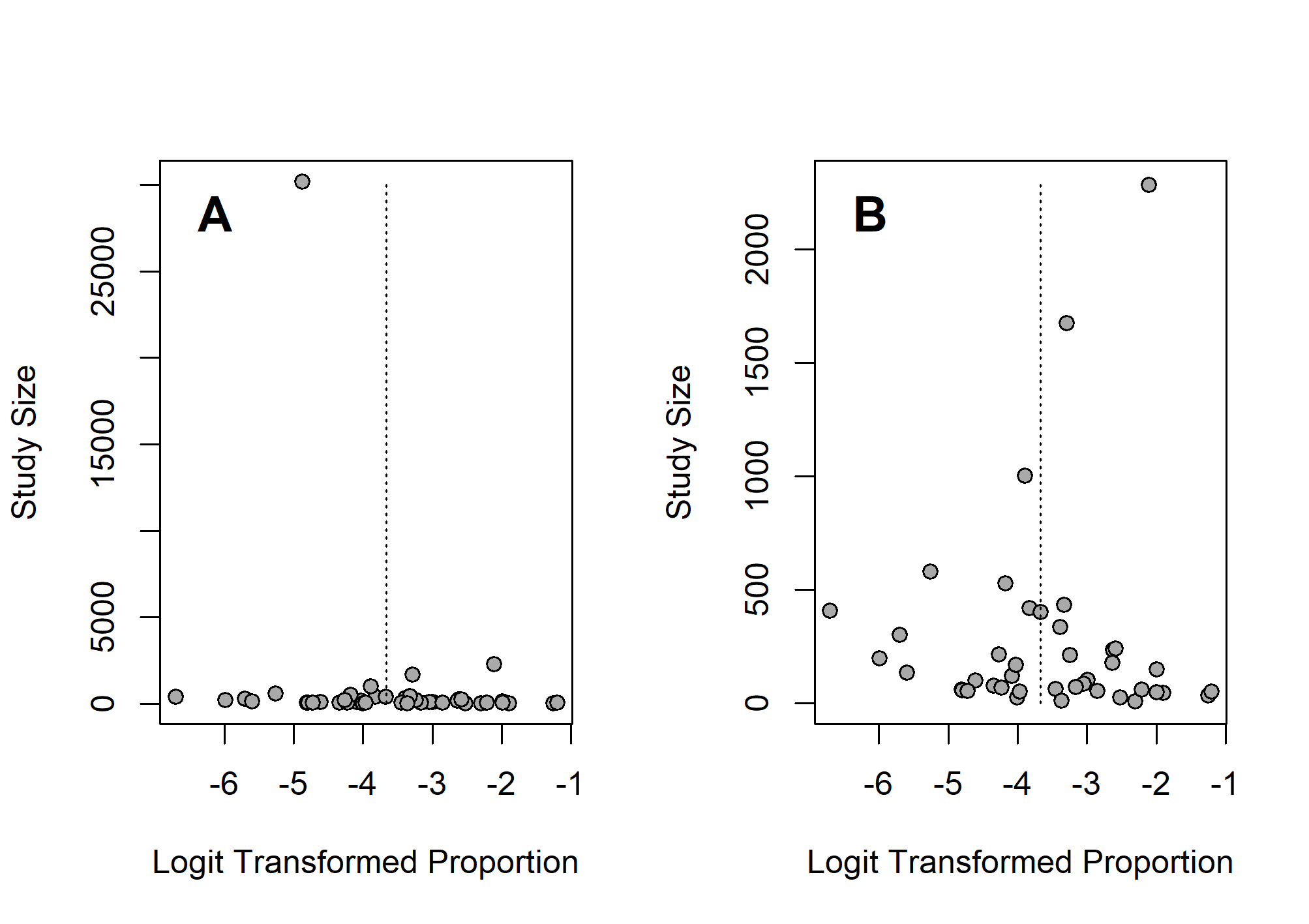


**Supplementary Figure 10: Funnel plot. (A)** Funnel plot of the logit transformed proportions versus the sample size for the respective proportion. The study conducted by Akinyemi and colleagues has an extreme sample size compared to the other studies included [A30]. **(B)** For visualisation purposes a new funnel plot is recreated excluding the study of Akinyemi and colleagues. The dotted line represents the estimated CFR on the logit scale.

When combining proportions, Hunter and colleagues recommend to plot the study size versus the logit transformed proportions [9]. The funnel plot in Supplementary Figure 10A shows that there is asymmetry, due to one study with an extreme sample size [A30]. We constructed the funnel plot once more, leaving out the study of Akinyemi and colleagues (Supplementary Figure 10B). In order to assess the asymmetry in a formal way, we used the test developed by Peters and colleagues [10]. This shows indeed that there is asymmetry (p-value= 0.0467). Supplementary Figure 10A shows a big gap between the study with the largest sample size and the remaining studies, the lack of studies being conducted with a large sample size, due to lack of resources in endemic countries, can serve as a possible explanation for the asymmetry. In the studies with smaller sample size (Supplementary Figure 10B), we observed quite some variation which might be attributable to heterogeneity, which can be explained by differences in methodological design, location, health care, etc. We tested for asymmetry excluding the study of Akinyemi and colleagues and found that there was no evidence for asymmetry (p-value= 0.397).

**References**

1. Higgins J, Altman D. Assessing risk of bias in included studies. In: Higgins J, Green S, eds. Cochrane Handbook for Systematic Reviews of Interventions. Wiley, 2011.

2. Rempel OR, Laupland KB. Surveillance for antimicrobial resistant organisms: potential sources and magnitude of bias. Epidemiol. Infect. **2009**; 137:1665–73. Available at: http://www.ncbi.nlm.nih.gov/pubmed/19493372.

3. Dettori JR. Loss to follow-up. Evid. Based. Spine. Care. J. **2011**; 2:7–10. Available at: http://www.pubmedcentral.nih.gov/articlerender.fcgi?artid=3427970&tool=pmcentrez&rendertype=abstract.

4. Mogasale V, Mogasale V V, Ramani E, et al. Revisiting typhoid fever surveillance in low and middle income countries: lessons from systematic literature review of population-based longitudinal studies. BMC Infect. Dis. **2016**; 16:35.

5. Devrim I, Ergunay K, Kara A, et al. The comparison of cultures, widal agglutination test and polymerase chain reaction as a diagnostic tool in typhoid fever. Cent. Eur. J. Med. **2008**; 3:470–474. Available at: http://ovidsp.ovid.com/ovidweb.cgi?T=JS&CSC=Y&NEWS=N&PAGE=fulltext&D=emed8&AN=2008498401%5Cnhttp://oxfordsfx.hosted.exlibrisgroup.com/oxford?sid=OVID:embase&id=pmid:&id=doi:10.2478/s11536-008-0052-8&issn=1895-1058&isbn=&volume=3&issue=4&spage=470&pages=47.

6. Andrews JR, Ryan ET. Diagnostics for invasive Salmonella infections: Current challenges and future directions. Vaccine **2015**; 33:C8–C15. Available at: http://linkinghub.elsevier.com/retrieve/pii/S0264410X1500208X.

7. Keddy K, Sooka A, Letsoalo M, et al. Sensitivity and specificity of typhoid fever rapid antibody tests for laboratory diagnosis at two sub-Saharan African sites. Bull. World Health Organ. **2011**; 89:640–647. Available at: http://www.who.int/bulletin/volumes/89/9/11-087627/en/.

8. AnkitRohatgi. WebPlotDigitizer. 2017; Available at: http://arohatgi.info/WebPlotDigitizer.

9. Hunter JP, Saratzis A, Sutton AJ, Boucher RH, Sayers RD, Bown MJ. In meta-analyses of proportion studies, funnel plots were found to be an inaccurate method of assessing publication bias. J. Clin. Epidemiol. **2014**; 67:897–903. Available at: http://dx.doi.org/10.1016/j.jclinepi.2014.03.003.

10. Peters JL, Sutton AJ, Jones DR, Abrams KR, Rushton L. Comparison of Two Methods to Detect Publication Bias in Meta-analysis. JAMA **2006**; 295:676. Available at: http://jama.jamanetwork.com/article.aspx?doi=10.1001/jama.295.6.676.
